# Multisensory processing of emotional cues predicts intrusive memories after virtual reality trauma

**DOI:** 10.1007/s10055-023-00784-1

**Published:** 2023-04-04

**Authors:** Naomi Heffer, Emma Dennie, Chris Ashwin, Karin Petrini, Anke Karl

**Affiliations:** 1 Department of Psychology, University of Bath, Claverton Down, Bath, BA2 7AY UK; 2 School of Sciences, Bath Spa University, Bath, UK; 3 Mood Disorders Centre, University of Exeter, Exeter, UK; 4 Centre for Applied Autism Research (CAAR), Bath, UK; 5 The Centre for the Analysis of Motion, Entertainment Research and Applications (CAMERA), Bath, UK

**Keywords:** Multisensory processing, Emotion recognition, Trauma film, Virtual reality, Intrusive memories

## Abstract

Research has shown that high trait anxiety can alter multisensory processing of threat cues (by amplifying integration of angry faces and voices); however, it remains unknown whether differences in multisensory processing play a role in the psychological response to trauma. This study examined the relationship between multisensory emotion processing and intrusive memories over seven days following exposure to an analogue trauma in a sample of 55 healthy young adults. We used an adapted version of the trauma film paradigm, where scenes showing a car accident trauma were presented using virtual reality, rather than a conventional 2D film. Multisensory processing was assessed prior to the trauma simulation using a forced choice emotion recognition paradigm with happy, sad and angry voice-only, face-only, audiovisual congruent (face and voice expressed matching emotions) and audiovisual incongruent expressions (face and voice expressed different emotions). We found that increased accuracy in recognising anger (but not happiness and sadness) in the audiovisual condition relative to the voice- and face-only conditions was associated with more intrusions following VR trauma. Despite previous results linking trait anxiety and intrusion development, no significant influence of trait anxiety on intrusion frequency was observed. Enhanced integration of threat-related information (i.e. angry faces and voices) could lead to overly threatening appraisals of stressful life events and result in greater intrusion development after trauma.

**Supplementary Information:**

The online version contains supplementary material available at 10.1007/s10055-023-00784-1.

## Introduction

When reading the emotional states of others, humans typically rely on multiple sensory inputs. These emotional cues include facial expression, voice prosody and body gesture. Multisensory emotion cues are processed interdependently, such that perception of emotion in faces is affected by accompanying voices and vice versa (De Gelder and Vroomen 2000; De Gelder et al. 2002). This is reflected in perceptual performance, as emotion judgements are generally quicker and more accurate for perception of emotion from audiovisual stimuli compared to visual or auditory stimuli presented in isolation (Collignon et al. 2008; Piwek et al. 2015). Therefore, a full ecological understanding of emotion and successful face-to-face interactions rely on effective multisensory processing (Klasen et al. 2012; Schirmer and Adolphs 2017).

Altered multisensory processing of emotional information has been observed in clinical populations including schizophrenia (De Jong et al. 2009), alcoholism (Maurage and Campanella 2013), autism spectrum disorders (Feldman et al. 2018) and anxiety (Koizumi et al. 2011; Yoon and Hong 2020; Heffer et al. 2021, 2022). Koizumi et al. (2011) showed that participants with higher trait anxiety were less accurate than participants with lower trait anxiety at recognising happy emotional cues when they were presented alongside angry cues. Furthermore, Heffer et al. (2022) showed that individuals with high trait anxiety were more likely to optimally integrate angry face and voice stimuli (i.e. threat-related stimuli) compared to individuals with low trait anxiety, while the same effect was not present for happy or sad stimuli. These findings suggest that multisensory emotion mechanisms in anxiety are optimised for processing threat, which could contribute to increased emotional reactivity to potentially threatening stimuli and therefore could possibly play a role in contributing to hypervigilance in anxiety- and stress-related disorders (such as PTSD).

One of the negative psychological outcomes which is associated with biased threat processing is the development of intrusive memories after experiencing a stressful life event (Mancini et al. 2021). Intrusive memories are unwanted, spontaneously occurring thoughts or mental images related to the stressful life event. Intrusive memories are not intentionally recalled but seem to come ‘out-of-the-blue’. Having intrusive memories about traumatic events is one of the core symptoms of PTSD and may manifest as unwanted upsetting memories, flashbacks or nightmares (APA 2013; Ehlers and Clark 2000; Ehlers and Steil 1995). While some cognitive processes that facilitate the development and maintenance of intrusive memories after trauma have been identified (James et al. 2016), nothing is currently known about the potential role of multisensory processing of emotional information for the formation of intrusive memories.

Established models of PTSD development propose that traumatic experiences are laid down as autobiographic memories when they are processed adaptively, in the form of a coherent narrative (Brewin 2001; Ehlers and Clark 2000; Ehlers and Steil 1995). However, maladaptive processing of trauma experiences results in fragmented memories which remain in a state of sensory vividness, where specific sensory features (e.g. noises, smells and images) are intensely re-experienced with a ‘here-and-now’ quality which causes a sense of persistent, current threat (Admon et al. 2013; Brewin 2001; Ehlers and Clark 2000). This vivid re-experiencing is considered to be a sign that memories of the event are poorly elaborated and inadequately integrated with relevant contextual information (Ehlers and Clark 2000; Ehlers and Steil 1995). It is possible that selective multisensory integration of threat-relevant information, such as that observed in high trait anxiety (Heffer et al. 2021; 2022), could lead to enhanced threat processing of stressful life events and result in increased intrusions (Mancini et al. 2021). However, an overall tendency towards more effective multisensory integration of emotional information could result in fewer intrusions, because memories will be better elaborated and integrated with contextual information and therefore will be less likely to be fragmented (Brewin 2001; Ehlers and Clark 2000).

Cognitive processes related to the development of intrusive memories have been studied using the trauma film paradigm (James et al. 2016). The trauma film paradigm involves showing film content depicting traumatic events to healthy, non-clinical samples. The artificial trauma simulation temporarily elicits psychophysiological responses that are similar to symptoms experienced after actual trauma, including intrusive memories of the traumatic event (James et al. 2016). This paradigm enables investigation of how peri-traumatic factors affect intrusive memory formation, in a way that is presumed to be analogous to the development of intrusive memories in early PTSD. The ecological validity of this paradigm has been enhanced in recent years by the use of new virtual reality (VR) technology to simulate more immersive trauma experiences (Baptie et al. 2021; Dibbets 2020; Nielsen et al. 2020).

Although empirical literature on the relationship between multisensory emotional processing and intrusive memory formation is virtually non-existent, there is existing evidence to suggest that more general differences in perception are likely to be important in predicting intrusive memories after trauma. Studies using the trauma film paradigm have shown that performing visuospatial tasks, such as playing the videogame ‘Tetris’, before, during or shortly after exposure to the trauma film, interferes with sensory-perceptual processing of the trauma and reduces subsequent intrusions (e.g. Holmes et al. 2009; Logan and O’Kearney 2012). By contrast, performing verbal tasks (e.g. completing a pub quiz) interferes with subsequent verbal and conceptual processing of the traumatic event, but not the vivid sensory features, and this serves to increase subsequent intrusions (e.g. Holmes et al. 2010; Nixon et al. 2007). These results support theories which suggest that persistence of intrusive memories following trauma results from enhanced sensory-perceptual processing, sometimes referred to as data-driven (or bottom-up) processing, at the expense of higher-order cognitive/contextual (or top-down) processing of traumatic events (Brewin 2001; Ehlers and Clark 2000; Kleim et al. 2012). This is relevant to the research questions in the current study because multisensory processing has been shown to involve modulations of both bottom-up and top-down perceptual mechanisms (De Gelder et al. 1999; Gao et al. 2018; Klasen et al. 2014).

It has also been shown that individuals with higher trait anxiety experience increased intrusions after the trauma film paradigm and that this relationship is mediated by peri-traumatic emotional processing (i.e. emotion dysregulation and anxious emotional response during or shortly after the trauma simulation) (Schweizer et al. 2017). Taken together, existing research using the trauma film paradigm in non-clinical samples to model the development of clinically relevant intrusive memories points to the important role of peri-traumatic emotional and perceptual processing for intrusion development, although the specific role of multisensory emotional processing has never been investigated.

The present study aimed to investigate the relationship between multisensory processing of emotional stimuli and the development of intrusive memories following exposure to a VR analogue trauma. Multisensory emotion recognition tasks were used to assess the ability of the participants to accurately recognise three core emotions (angry, sad and happy) from audio, visual and audiovisual face and voice stimuli. Owing to the dearth of previous research on the link between multisensory processing and intrusive memory development, the current study was largely exploratory. Different aspects of multisensory performance were assessed using different variations of the emotion recognition paradigm. A directed version of the task, where participants were instructed to base their judgements specifically on either the auditory or visual information, was used to measure crossmodal interference, which is a measure of the perceptual interference caused by incongruent emotional information. Based on previous research showing higher crossmodal interference for individuals with high trait anxiety when attempting to ignore irrelevant threat-related cues (Koizumi et al. 2011), we hypothesised that higher crossmodal interference for stimuli involving angry cues might predict more intrusions.

Participants also completed an undirected version of the emotion recognition task where they were instructed to base their judgements on their overall impression of the stimuli, rather than paying attention to cues in a specific modality. This task was used to measure accuracy in judging emotion from audiovisual stimuli, as well as potential biases towards negative and/or threatening interpretations of ambiguous stimuli, and a measure of multisensory integration ability. Based on previous research in individuals with high trait anxiety (Koizumi et al. 2011; Heffer et al. 2021, 2022), we expected that selectively enhanced multisensory integration of threat-relevant information would result in increased intrusions, but that an overall tendency towards more effective multisensory integration of emotional information would result in fewer intrusions, due to better elaboration of trauma memories as a result of more effective integration.

A unimodal task where participants judged emotion from faces and voices presented in isolation was also used to as a control measure to allow us to discern whether any observed effects of multisensory processes were specific to multisensory perception and not attributable to more generalised differences in emotion perception. We also measured trait anxiety to control for the effects of trait anxiety on intrusion development and multisensory processing, as we predicted based on previous research that higher trait anxiety would be associated with more intrusions (Schweizer et al. 2017) and because previous research has shown that trait anxiety affects multisensory emotion processing by enhancing multisensory processing of threat-related cues.

## Methods and materials

### Participants

Participants were 57 students (45 *F*/11 *M*/1 undisclosed) from the University of Exeter (age: *M* = 18.96, SD = 0.95) who were recruited from a larger sample of 135 volunteers who filled in an online screening questionnaire. Of the 57 participants who completed the laboratory session of the study, 55 were included in the analyses (see Appendix A of Online Resource 1 for participant flowchart). One participant was excluded from the analyses because the diary measure was not returned at the end of the 7-day measurement period. One additional participant was excluded due to missing task data in some conditions of the multisensory emotion recognition task which was the result of a technical issue.

The target sample size of 80 participants was determined by an a priori power calculation for multiple regression with three predictors to detect a medium effect size (*f*^2^ = 0.15) with 80% power (*p* = 0.05), which is consistent with the effect sizes typically reported in studies using the trauma film paradigm to model intrusive memory development (Clark et al. 2015). The planned study involved measuring more than three potential predictors; however, the choice to base the power calculation on a regression with only three predictors was based on an estimate of how many important predictors we thought would be likely to end up in the regression models following entry using stepwise sequential methods (described in more detail in Sect. 2.4 Statistical Analysis). This estimate was based on previous research in trait anxiety which has shown that of the many multisensory processes that have been investigated, only a small number have been found to be significantly related to trait anxiety levels (Koizumi et al. 2011; Heffer et al. 2021, 2022). We were unable to recruit to target because of COVID-related laboratory closures in March 2020, which meant that the attained sample was only sufficiently powered to detect medium-to-large effects.

To be included in the present study, participants had to be 18 years or older and report normal, or corrected-to-normal, vision and hearing. Exclusion criteria were used to ensure that volunteers who were more likely to be excessively distressed by the trauma simulation were excluded. Individuals were excluded if they reported having previously been in a serious car accident, if they had a previous diagnosis of a mental health condition (as self-reported by participants), current low mood (as indicated by a score of ten or more on the Patient Health Questionnaire depression scale, PHQ-8; Kroenke et al. 2009) or if they had current symptoms of PTSD (as indicated by a score of three or more on the Primary Care PTSD Screen for DSM-5, PC-PTSD-5; Prins et al. 2016). Because the VR trauma induction paradigm used in this study was also used as part of a different study involving measurement of physiological parameters, including heart rate, participants were also excluded if they reported having current or existing heart problems or a pacemaker. We do not report on these physiological measures here because they were not relevant to the research questions being asked in this study. Participants were also excluded if they reported a diagnosis of epilepsy, as some forms of epilepsy can be triggered by intense visual stimulation. Participants were compensated with £10 or course credits for their time.

### Materials

#### Self-report questionnaires

The trait subscale of the Spielberger State-Trait Anxiety Inventory (STAI-T, Y-Form; Spielberger, 1983) was used to measure anxious apprehension. The Anxiety Sensitivity Index (ASI-3) (Taylor et al. 2007) was included as an additional anxiety measure because it has been recommended as a useful measure to isolate ‘anxious arousal’ processes from ‘anxious apprehension’ processes (Nitschke et al. 2001). More information about the scales and their psychometric properties is found in Appendix A of Online Resource 1.

Participants rated their level of current stress on a scale of 0–100 in response to the question “How stressed do you feel at the moment?” Participants gave a stress rating at the start of the laboratory session and then again immediately after the VR simulation. These measurements were used to test the effectiveness of the VR simulation at inducing psychological distress.

#### VR analogue trauma simulation

**Fig. 1:**
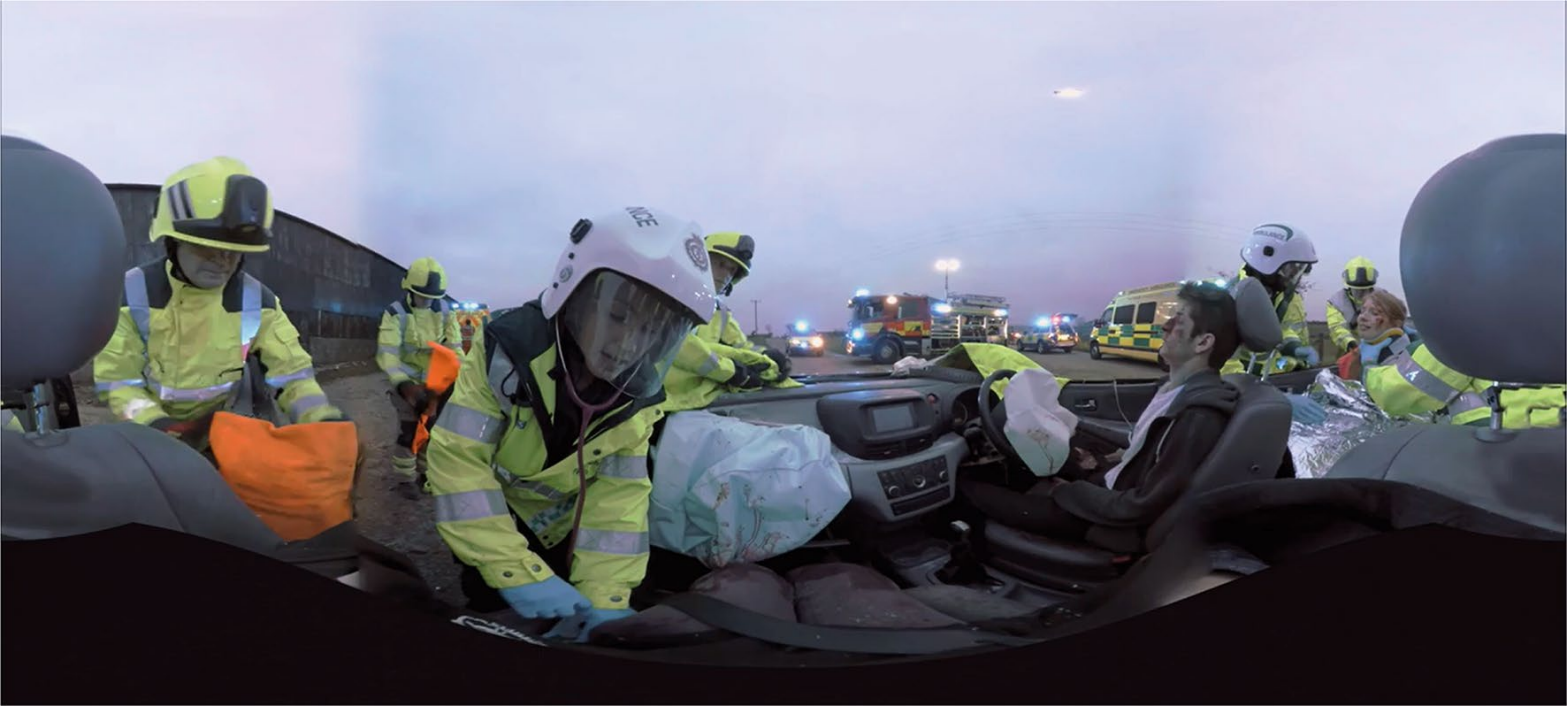
Scene from VR trauma simulation

Participants experienced the audiovisual VR simulation via an HTC VIVE VR headset. The VR scene depicted a car accident from the perspective of the passenger sitting next to the driver. Following the accident, the participant experienced scenes of paramedics and rescue personnel trying to help the injured passengers out of the wrecked car, as shown in Fig. 1. This was an audiovisual simulation, consisting of both social and non-social audio and visual emotion cues, including fearful facial expressions and verbal cues from the passengers, and visual scenes of the car wreckage, as well as emotive sounds such as crunching metal and sirens. The film lasted for approximately 6.5 min and was produced by FirstCar & Leicestershire Fire and Rescue Service (2017) to raise awareness about the dangers of driving while distracted among young people. Consistent with previous studies that have used similar films depicting road traffic accidents to study intrusion development, this film was used with the intention of inducing a state of temporary distress. Previous studies using the paradigm have reported no serious or prolonged distress in participants (e.g. James et al. 2016; Weidmann et al. 2009).

#### Multisensory emotion recognition task

The multisensory emotion recognition task required participants to make a judgement about the emotion expressed in a series of face and voice stimuli which expressed either happy, sad or angry emotions. The stimuli were presented in four different modality conditions: visual-only, audio-only, audiovisual congruent and audiovisual incongruent. Participants sat approximately 57 cm from a computer screen on which the visual stimuli were presented and auditory stimuli were binaurally presented through over-the-ear headphones. On each trial, participants were presented with a fixation point for 1000 ms followed by the stimulus. Responses were made by a speeded key press with the dominant hand. Participants were asked to categorise stimuli as quickly as possible, but not to the detriment of accuracy. There were three different response options (i.e. happy, sad and angry) and so three different keys used for responding to stimuli. There were six possible permutations of emotion-key assignments for responding to stimuli; these were randomised across participants.

Stimuli were 500 ms-long videos of female adults vocalising the phrase ‘the door’ in a manner which was angry, happy or sad. Videos were adapted from longer clips taken from the RAVEDESS database (Livingstone and Russo 2018a, 2018b), as described in a previous research study (Heffer et al. 2022). A total of 90 stimuli were used in the tasks and were produced from clips of six female actors portraying three different emotions. There were 18 stimuli for each emotion (e.g. visual-only, audio-only and audiovisual congruent stimuli) and 36 audiovisual incongruent stimuli, made up from all possible combinations of face and voice cues in the different emotions.

Participants completed four variations of the task: the ‘audio-directed attention task’, the ‘visual-directed attention task’, the ‘undirected attention task’ and the ‘unimodal task’. In the directed attention tasks, participants were presented with audiovisual congruent and audiovisual incongruent stimuli and were instructed to make their judgement based on just the faces while ignoring the accompanying voices, or vice versa. In the ‘undirected attention task’, participants were again shown audiovisual congruent and incongruent stimuli, but in this variation of the task they were asked to give their ‘overall impression’ of the stimuli, i.e. they could use both face and voice information when making their judgements. In the ‘unimodal task’, participants were required to make judgements about the emotions shown in audio-only and visual-only stimuli. The order in which the different task conditions were completed was randomised for each participant. The audio-directed, visual-directed and undirected attention tasks each consisted of 162 trials presented in three blocks of 54 trials. The unimodal task consisted of 108 trials presented in three blocks of 36 trials.

Behavioural outcomes from the multisensory emotion recognition task, which were entered as predictor variables into the regression models, are summarised in Table 1.

**Table 1:** Behavioural outcome measures from the multisensory emotion recognition task

	Outcome	Task	Construct measured	Calculation
1	Audio accuracy	Unimodal	Accuracy recognising emotion from voices	% Accuracy for audio-only trials
2	Visual accuracy	Unimodal	Accuracy recognising emotion from faces	% Accuracy for visual-only trials
3	Audiovisual accuracy	Undirected	Accuracy recognising emotion from congruent face and voice pairs	% Accuracy for audiovisual congruent trials
4	Happy accuracy	Unimodal/undirected	Accuracy for happy stimuli	% Accuracy for happy trials
5	Angry accuracy	Unimodal/undirected	Accuracy for angry stimuli	% Accuracy for angry trials
6	Sad accuracy	Unimodal/undirected	Accuracy for sad stimuli	% Accuracy for sad trials
7	Overall multisensory facilitation score	Unimodal/undirected	Perceptual benefit through multisensory integration of congruent face and voice cues	Difference between % accuracy for audiovisual congruent trials and the best unimodal condition (Meredith & Stein 1983)
8	Multisensory facilitation score for happy stimuli	Unimodal/undirected	Perceptual benefit through multisensory integration of happy face and voice cues	Difference between % accuracy for happy audiovisual congruent trials and the best unimodal condition for happy stimuli
9	Multisensory facilitation score for angry stimuli	Unimodal/undirected	Perceptual benefit through multisensory integration of angry face and voice cues	Difference between % accuracy for angry audiovisual congruent trials and the best unimodal condition for angry stimuli
10	Multisensory facilitation score for sad stimuli	Unimodal/undirected	Perceptual benefit through multisensory integration of sad face and voice cues	Difference between % accuracy for sad audiovisual congruent trials and the best unimodal condition for sad stimuli
11	Multisensory threat bias	Undirected	Tendency to interpret incongruent face and voice displays as angry	% ‘angry’ judgements on audiovisual incongruent trials minus the expected percentage for a neutral observer (i.e. 33.33%). A score of 0 indicates no bias, positive scores indicate a bias towards threat, and negative scores indicate a bias away from threat
12	Multisensory negativity bias	Undirected	Tendency to interpret incongruent face and voice displays as negative (angry or sad)	% ‘sad’ and ‘angry’ judgements on audiovisual incongruent trials minus the expected percentage for a neutral observer (i.e. 66.67%). A score of 0 indicates no bias, positive scores indicate a bias towards negative information, and negative scores indicate a bias away from negative information
13	Overall crossmodal interference	Directed	The level of perceptual interference caused by to-be-ignored face or voice cues in audiovisual incongruent displays	Accuracy on audiovisual congruent trials minus accuracy on audiovisual incongruent trials
14	Happy crossmodal interference	Directed	The level of perceptual interference caused by to-be-ignored happy face or voice cues in audiovisual incongruent displays	Accuracy on audiovisual congruent trials minus accuracy on audiovisual incongruent trials containing to-be-ignored happy cues
15	Angry crossmodal interference	Directed	The level of perceptual interference caused by to-be-ignored angry face or voice cues in audiovisual incongruent displays	Accuracy on audiovisual congruent trials minus accuracy on audiovisual incongruent trials containing to-be-ignored angry cues
16	Sad crossmodal interference	Directed	The level of perceptual interference caused by to-be-ignored sad face or voice cues in audiovisual incongruent displays	Accuracy on audiovisual congruent trials minus accuracy on audiovisual incongruent trials containing to-be-ignored sad cues

We calculated accuracy scores, i.e. percentage of correct responses, separately for audio, visual and audiovisual congruent stimuli and for happy, angry and sad stimuli. We also calculated a measure of multisensory integration ability termed a ‘Multisensory Facilitation Score’, which was calculated as the difference between accuracy for audiovisual congruent trials on the undirected attention task and accuracy for the best unimodal condition on the unimodal task (Meredith and Stein 1983). The formula used for this calculation was: $\text{Multisensory Facilitation}=p\left(AV\right)-\mathrm{max}\left\{p\left(A\right), p\left(V\right)\right\}$, where *p* is the percentage of correct responses in each condition. A positive Multisensory Facilitation score indicated better performance in audiovisual congruent trials compared to unimodal trials, while a negative score indicated better performance in unimodal trials than audiovisual congruent trials.

For audiovisual incongruent trials in the undirected attention task, it was not possible to measure the accuracy of emotion recognition because in these incongruent displays, multiple emotions were represented in the visual and auditory information and so there was no ‘correct’ emotion judgement. Therefore, rather than analysing accuracy for incongruent stimuli in this task, the tendency to respond angry (multisensory threat bias) or negatively (i.e. angry or sad) (multisensory negativity bias) was calculated instead. These bias scores were calculated by finding the percentage of ‘angry’ or ‘angry and sad’ judgements on audiovisual incongruent trials of the undirected attention task and then subtracting the expected percentage of angry/sad responses for a neutral observer (i.e. an observer who had a 1 in 3 chance of picking each of the emotions), as explained in more detail in Table 1. A score of 0 indicated no bias, positive scores indicated a bias towards interpreting displays as threatening/negative, and negative scores indicated a bias away from interpreting displays as threatening/negative. The use of this parameter in the analysis is in line with the methods of a number of relevant studies (Collignon et al. 2008; Piwek et al. 2015; Heffer et al. 2021). This was a measure of emotional, rather than attentional, bias, as not all emotions were represented in all incongruent stimuli. However, existing multisensory research has shown that when incongruent emotional cues are integrated together, an emotional McGurk effect may occur, resulting in an entirely new percept (Fagel 2006). This means, for example, that even for incongruent stimuli comprising angry and happy cues only, participants may still make an overall judgement of ‘sad’, even though this emotion was not present in either the auditory or the visual cue. A summary of mislabelling frequencies, showing evidence of an emotional McGurk effect in participants’ judgements of audiovisual incongruent stimuli, is presented in Appendix B (Table B6).

We also calculated ‘crossmodal interference’ from the directed attention tasks, which is a measure of the level of perceptual interference caused by to-be-ignored face or voice cues in audiovisual incongruent displays. To calculate crossmodal interference from the data in the directed attention tasks, the percentage accuracy in audiovisual incongruent trials was subtracted from the percentage accuracy in audiovisual congruent trials for each participant. Higher crossmodal interference scores indicated that processing of incongruent information in the to-be-ignored modality was more likely to interfere with perception of emotion in the attended modality. Crossmodal interference was calculated overall across all stimulus emotions, and then separately for the three emotions, i.e. happy crossmodal interference refers to the level of perceptual interference when to-be-ignored happy face or voice cues were competing for attention in audiovisual incongruent displays.

#### Diary measure

Participants were given a paper diary to record intrusive thoughts and images related to the analogue trauma over the seven days immediately following the VR simulation. For each memory recorded, they reported the day that it occurred (i.e. day 1–7 following the analogue trauma), a brief sentence summarising the content of the intrusive memory and then a rating of their emotional distress in response to the intrusion (“How distressed were you at the intrusion? 0 not at all, 10 extremely”). Participants were instructed to record only those memories of the trauma film which occurred spontaneously, as opposed to thoughts which they had when deliberately recalling their participation in the study. Participants were encouraged to carry the diary measure with them for the seven days following the study so that they could record the intrusive memory in their diary as soon as possible after it occurred. If no intrusive memories occurred on a particular day during the seven-day period, participants were asked to record a zero in their diary for that day. Similar seven-day diary measures have been widely used in studies of the trauma film paradigm to assess the frequency and level of distress associated with intrusions (James et al. 2016).

Aside from the one participant who did not return the diary measure at the end of the seven-day period, and so was not included in the analyses reported in this paper, there were no missing datapoints for the diary measure. We calculated intrusion frequency as the total number of intrusive memories recorded using the seven-day diary measure. In the present study, the number of intrusions over seven days ranged from 0 to 16 (*M* = 3.49, SD = 3.92). The mean level of distress for each of the seven days was calculated by summing the distress ratings for all intrusions reported on any given day and then dividing by the number of intrusions reported for that day. Days on which no intrusions were recorded had a mean distress score of zero. The level of mean daily distress over seven days was then calculated by adding up the mean distress ratings for each day and dividing this by seven (i.e. the number of days for which the diary measure was administered). Mean daily distress ratings were calculated in this way to be more representative of the overall level of distress experienced by individuals following the trauma simulation, i.e. so that an individual with a small number of very distressing intrusions did not end up with a disproportionately large distress score compared to an individual who had many moderately distressing intrusions. For individuals experiencing intrusions, the mean level of daily distress related to intrusions ranged from 0 to 8.71 out of a possible score of 10 (*M* = 1.34, SD = 1.55). Histograms showing the overall results for the diary measure are presented in Appendices C and D of Online Resource 1.

### Procedure

Participants first completed the online questionnaire measures (STAI-T and ASI-3) and were then invited to sign up for a time to participate in the laboratory portion of the study. Participants were tested individually in a quiet laboratory space at the University of Exeter. On arrival, participants gave a rating of their current stress levels and then participated in the multisensory emotion recognition task. Before starting the task, participants completed three practice trials to check they understood the task and were happy to proceed. After completion of the multisensory emotion recognition task, participants were offered a short break (approximately five minutes) before they were then exposed to the VR simulation. After the VR simulation, they gave a second rating of their stress levels. Overall, the laboratory tasks took approximately 50 min to complete. Before leaving the laboratory, the experimenter explained the instructions for the diary task and then participants completed a guided mood-repair activity where they were asked to imagine a pleasant past experience. This activity was carried out as part of the aftercare procedures to ensure that if participants felt distressed following the VR simulation, some of this distress was alleviated before leaving the laboratory. Participants completed the diary measure everyday over the following seven days and then returned to the laboratory after seven days to hand in the paper diary.

### Statistical analysis

#### Software packages

Data were analysed using R version 3.6.2 (R Core Team 2019). R packages used for analysis included the MASS package (Venables et al. 2002), the pscl package (Zeileis et al. 2008), the AER package (Kleiber and Zeileis 2008), the ggplot2 package (Wickham 2016), the boot package (Canty 2002) and the betareg package (Cribari-Neto and Zeileis 2010).

#### Data preparation and model assumptions

Given that there were many behavioural measures, a regression procedure with a stepwise (sequential) entry criterion was chosen. This stringent procedure for entering regressors into the model was used to ensure that only a small subset of predictors was tested at any given time, increasing our statistical power to detect effects for the most influential predictors.

The intrusion frequency consisted of count data, so initially, a Poisson distribution was selected to model the data. However, an important assumption of the Poisson distribution is that the mean and variance are equal. In order to determine whether this assumption was met, we carried out a test to look for potential overdispersion of the data, which revealed that the variance of the intrusion frequency data was significantly larger than the mean, *Z* = 2.70, *p* = 0.003, and so indicated that the outcome data were over-dispersed (Cameron and Trivedi 1990). For over-dispersed count data, a negative binomial model is considered more appropriate than a Poisson (Long 1997). Therefore, a negative binomial model was applied to analyse the intrusion frequency data.

Intrusion frequency was analysed for all 55 participants, but distress related to intrusions was only analysed for those individuals who reported experiencing at least one intrusion over the seven days following the trauma film (*n* = 41). For each of the seven days, participants had a possible distress score of between 0 and 10, so by considering the level of mean daily distress over seven days, we effectively ended up with a proportion out of 70 (we obtained this proportion by dividing our ‘mean daily distress’ variable by 10). We opted to analyse the data using a beta distribution, as this type of model is ideal for proportional data (Ferrari and Cribari-Neto 2004). After dividing mean daily distress ratings by 10 to obtain proportions, scores were further transformed by applying the formula: $y=(y\cdot \left(n-1\right)+0.5)/n$ (Smithson and Verkuilen 2006), which ensured that there were no exact zeros or ones in the outcome data, as these are not compatible with the betareg package used to run the regression model (Cribari-Neto and Zeileis 2010).

#### Statistical design

Two different stepwise regressions were used to explore the relationship between the independent variables (i.e. STAI-T scores (anxious apprehension), ASI-3 scores (anxious arousal), emotion recognition accuracy scores, multisensory facilitation scores, multisensory threat/negativity bias and crossmodal interference) and the frequency and distress related to intrusive memories over the seven days following testing. Stepwise models have been applied in previous studies using the trauma film paradigm to investigate potential predictors of subsequent intrusion development (Dibbets 2020).

## Results

Descriptive statistics relating to participant performance on the multisensory emotion tasks and a correlation matrix showing the relationships between all predictor variables is found in Appendix B of Online Resource 1.

### Manipulation check

To test the effectiveness of the VR simulation at inducing psychological stress, a paired Wilcoxon signed-rank test was used to determine whether there was a significant difference between pre-VR (*M* = 15.50; SD = 15.54) and post-VR (*M* = 36.50; SD = 20.90) stress ratings. A nonparametric test was used as the stress scores were not normally distributed. The results revealed that the stress scores after the VR trauma were significantly higher compared to stress scores at the start of the testing session, *z* = 6.08, *p* < 0.001, showing that the VR simulation was effective in increasing the stress levels of the participants. Three individuals failed to provide pre-VR stress ratings, so these statistics are based on data from 52 cases.

### Intrusion frequency

**Fig. 2:**
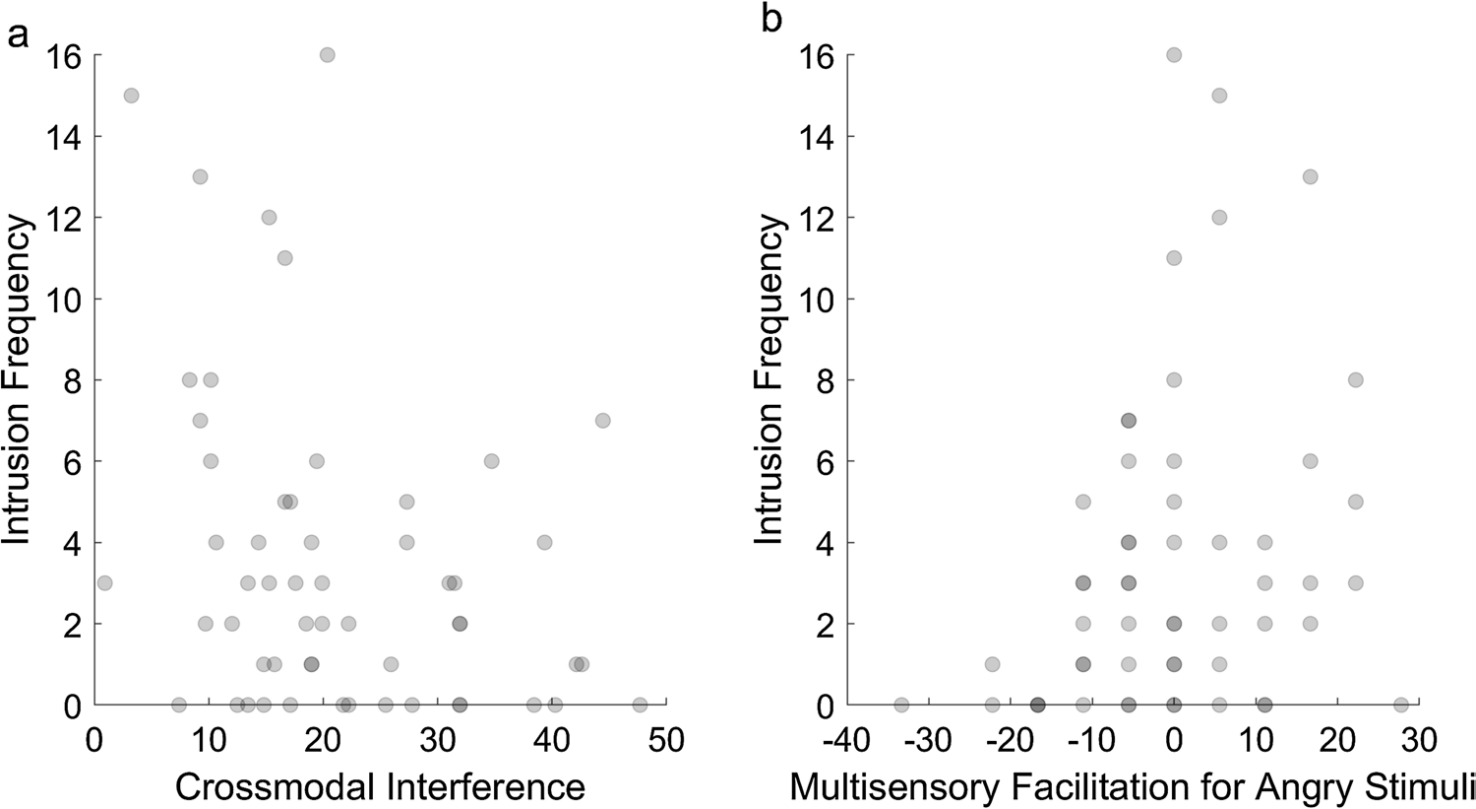
The relationship between intrusion frequency and the two significant predictors in the stepwise negative binomial regression model: **a** Crossmodal interference and **b** multisensory facilitation for angry stimuli. The darker circles indicate that multiple participants had the same value

The stepwise procedures produced the model that was the best fit for the data and which was significantly better than the null (intercept-only) model, *G*^2^ (3) = 12.79, *p* = 0.005. Table 2 shows the predictors which were included in the model at each stage of the stepwise procedures. Crossmodal interference was a significant negative predictor of the number of intrusions, *b* = − 3.75, 95% CI [− 6.49, − 1.09], SE = 1.41, *z* = − 2.67, *p* = 0.008, as shown in Fig. 2a. Specifically, an increase of one standard deviation from the mean in the size of the crossmodal interference effect (*M* = 21.47, SD = 11.07) predicted a 1.03 decrease in the number of intrusions. The multisensory facilitation score for angry stimuli was a significant positive predictor of the number of intrusions, *b* = 2.90, 95% CI [0.23, 5.67], SE = 1.18, *z* = 2.46, *p* = 0.014, as shown in Fig. 2b. Specifically, an increase of one standard deviation from the mean in terms of the multisensory facilitation score for angry stimuli (*M* = − 0.30, SD = 12.40) predicted a 1.31 increase in the number of intrusions. Accuracy in interpreting emotion from sad stimuli was another positive predictor which appeared in the final model, although it did not significantly predict intrusion frequency, *b* = 1.58, 95% CI [− 0.46, 3.64], SE = 1.01, *z* = 1.57, *p* = 0.115. Residual plots and statistics relating to model fit are reported in Appendix C of Online Resource 1.

**Table 2:** Stepwise regression model with intrusion frequency as the dependent variable

Step	Variable	2LL	*b*	SE	*Β*	*P*
1	Constant	− 256.43	1.20	0.15		< .001
	Angry multisensory facilitation score		3.51	1.25	0.11	.005
2	Constant	− 251.36	1.80	0.31		< .001
	Angry multisensory facilitation score		3.14	1.18	0.10	.008
	Crossmodal interference		− 3.05	1.36	− 0.09	.025
3	Constant	− 249.80	0.80	0.72		.264
	Angry multisensory facilitation score		2.90	1.18	0.09	.014
	Crossmodal interference		− 3.75	1.41	− 0.11	.008
	sad accuracy		1.58	1.01	0.06	.115

2LL = 2 × log likelihood

### Distress related to intrusions

**Fig. 3:**
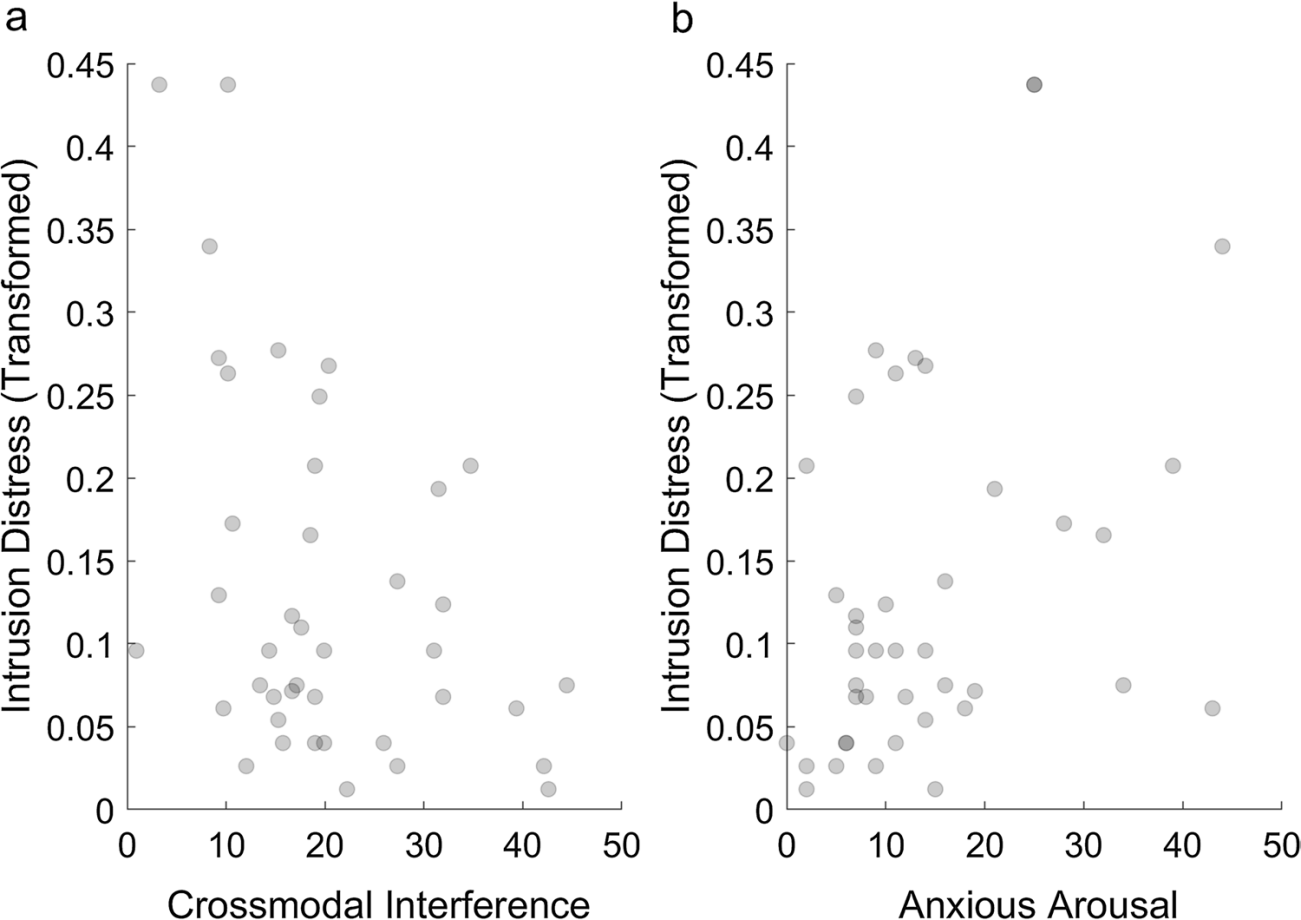
The relationship between intrusion-related distress ratings (transformed for the purposes of the analysis, see Methods for more information) and the two predictors in the stepwise beta regression model: **a** crossmodal interference and **b** anxious arousal (ASI-3 score). Note that neither predictor was significant when using bootstrapped estimates to account for the lack of normality in the obtained residuals. The darker circles indicate that multiple participants had the same value

The first iteration of the model revealed one case with a large Cook’s distance (> 0.5), suggesting that this case was exerting undue influence on the results of the regression; therefore, the model was re-run while winsorizing this case (replacing the outcome value with the next most extreme value in the dataset); see Appendix D of Online Resource 1 for more information. The stepwise procedures produced the model that was the best fit for the data and was significantly better than the null (intercept-only) model, *χ*^2^ (2) = 11.62, *p* = 0.003. Similar to the result for intrusion frequency, crossmodal interference was found to be a negative predictor of the level of distress related to intrusions, *b* = − 2.66, 95% CI [− 4.82, − 0.50], SE = 1.10, *z* = − 2.41, *p* = 0.016, as shown in Fig. 3a. Anxious arousal (ASI-3 score) was a positive predictor of distress related to intrusions, *b* = 0.02, 95% CI [0.004, 0.04], SE = 0.01, *z* = 2.39, *p* = 0.017, as shown in Fig. 3b.

Table 3 shows the predictors which were included in the model at each stage of the stepwise procedures. Subsequent plotting of the model residuals suggested that residuals were not normally distributed, and so bootstrapped confidence intervals for the coefficients in the final model were also calculated. Bootstrapped confidence intervals (BCI) suggested that both predictors failed to reach significance: crossmodal interference 95% BCI [− 4.73, 0.07] and anxious arousal 95% BCI [− 0.003, 0.042].

**Table 3:** Stepwise regression model with distress related to intrusions as the dependent variable

Step	Variable	Pseudo *R*^*2*^	*b*	SE	*p*
1	Constant	0.17	− 1.29	0.24	< .001
	Crossmodal interference		− 3.03	1.15	.008
2	Constant	0.28	− 1.71	0.30	< .001
	Crossmodal interference		− 2.66	1.10	.016
	Anxious arousal (ASI-3 score)		0.02	0.01	.017

Adding an interaction term between anxious arousal and crossmodal interference did not significantly improve the fit of the model, *χ*^2^ (1) < 0.01, *p* = 0.957, and the interaction term itself was not a significant predictor, *b* = − 0.01, SE = 0.10, *z* = − 0.10, *p* = 0.956, which suggests that any potential effects of crossmodal interference and anxious arousal on intrusion-related distress are likely to be independent.

Residual plots and statistics relating to model fit are reported in Appendix D of Online Resource 1.

## Discussion

Our findings demonstrate for the first time an association between individual differences in multisensory processing of emotion cues and the frequency of intrusions after a simulated traumatic event. The results showed that enhanced multisensory processing of angry faces and voices, but not sad or happy cues, was associated with a greater number of intrusions following a VR analogue trauma experience. We also found that increased perceptual interference resulting from processing of irrelevant face and voice cues was associated with both lower frequency of intrusive memories and less distress elicited by intrusions. Together, the results showed that enhanced multisensory processing of threat in faces and voices and reduced susceptibility to distraction by irrelevant emotional information were related to a greater frequency of intrusive memories, and so may contribute to a less adaptive psychological response to trauma.

The findings showed that enhanced multisensory processing of threat from faces and voices predicted more intrusions after induced trauma. This result was in line with our predictions based on existing research in high trait anxiety, which has shown that individuals with high anxiety levels show enhanced multisensory facilitation effects for angry faces and voices (Heffer et al. 2021, 2022). In the current study, participants who showed greater accuracy for angry stimuli in the audiovisual congruent condition compared to the unimodal (i.e. audio- and visual-only) conditions reported a higher number of intrusive memories of the analogue trauma event over the subsequent seven days. These participants had higher multisensory facilitation scores for angry stimuli, indicating that they were better able to make use of the additional information in audiovisual displays to detect anger.

Contrary to the findings of previous studies (Collignon et al. 2008; Piwek et al. 2015), although participants were significantly more accurate at recognising the stimulus emotion for audiovisual congruent stimuli compared visual-only stimuli, there was no significant difference between accuracy in the audio-only condition and the audiovisual congruent condition. This meant that some participants had a negative multisensory facilitation score, indicating that they performed better with just one of the senses compared to two. For angry stimuli, the mean multisensory facilitation score was close to 0 (*M* = − 0.30). Analysis of the unimodal trials indicates that the voices were recognised significantly more reliably than the faces. The extent of multisensory integration has been shown to be dependent on the relative reliability of the involved sensory cues, with greater multisensory facilitation effects occurring when the different senses are similarly reliable (e.g. Ernst and Banks 2002). Owing to the significantly superior reliability of the voice cues in the face and voice stimuli, the less reliable visual information may have been distracting for some individuals, rather than helpful, when judging emotion from the audiovisual stimuli. Individuals who experienced more intrusions, however, were those who had higher multisensory facilitation scores for angry stimuli; in other words, those who were not negatively distracted or affected by the less reliable visual information or showed a further benefit from having this information when recognising the portrayed emotion. This supports our suggestion that individuals who experienced more intrusions were those who were able to process multisensory threat-related information more efficiently compared to other individuals, even when information in one modality was less reliable and possibly distracting.

The tendency towards enhanced integration of threat-related emotional cues relative to other types of emotional information may serve to exhaust capacity for processing of other emotional stimuli, preventing adaptive elaboration of trauma memories (Ehlers and Clark 2000; Ehlers and Steil 1995) and contributing to increased emotional reactivity to potentially threatening stimuli, resulting in increased hyperarousal and intrusions following trauma (Mancini et al. 2021). Given the evidence to suggest that a tendency towards enhanced integration of threat cues may be associated with both high trait anxiety and increased intrusions after trauma (Heffer et al. 2021; 2022), future studies may consider using a more highly powered sample to test for a moderating influence of trait anxiety on the relationship between multisensory facilitation for threat-related stimuli and intrusive memories after trauma.

As well as the anger-specific effect observed in relation to multisensory processing of threat cues, we also observed an emotion-general effect of crossmodal interference. This suggests that in contrast to high trait anxiety, where only enhanced multisensory processing of threat has been linked to differences in psychological distress (Heffer et al. 2021, 2022; Koizumi et al. 2011), individual differences in multisensory processing of other emotions may be important in predicting the frequency of intrusions following trauma. Greater crossmodal interference in the multisensory emotion recognition tasks was associated with fewer intrusive memories and reduced levels of psychological distress elicited by intrusions. Crossmodal interference is a measure of the perceptual interference which occurs when conflicting information is presented in different sensory channels, and the ability to filter out irrelevant crossmodal information is thought to be indicative of better executive control (Araneda et al. 2015; Hirst et al. 2019; Spagna et al. 2020). Contrary to previous research which suggests that poor cognitive or executive control predicts increased intrusions following the trauma film paradigm (Verwoerd et al. 2011), we found that participants with larger crossmodal interference effects had significantly fewer intrusive memories and less distressing intrusions.

One potential explanation for this apparent discrepancy is that individuals with high crossmodal interference scores on this task are those who processed more irrelevant sensory-perceptual information shortly before exposure to the trauma film. Previous studies have shown that completing visuospatial tasks, like playing ‘Tetris’, before, during or shortly after trauma exposure leads to competition for the sensory-perceptual resources needed to generate intrusive memories, resulting in fewer intrusions (e.g. Holmes et al. 2009; Logan and O’Kearney 2012). In the current study, the additional irrelevant information processed by participants with high crossmodal interference scores may have interfered with subsequent sensory-perceptual processing of the analogue trauma simulation, reducing subsequent intrusions.

In the current study, we observed effects of our multisensory predictors in the absence of effects for processing of single sensory cues. For example, enhanced multisensory processing of angry faces and voices was associated with higher frequency of intrusions, but overall accuracy on angry trials or audio-/visual-only trials was not significantly associated with the frequency of intrusions. This suggests that differences in intrusion development were specifically related to multisensory processing of emotional information (i.e. how audio and visual cues were combined), rather than more general differences in sensory processing, and that differences in multisensory processing may be a more sensitive biomarker than differences in processing information from single senses when predicting an individual’s emotional response to trauma. This is consistent with previous research showing that brain activity underlying the perception of audiovisual emotional stimuli is a more sensitive biomarker of subclinical anxious and depressive tendencies than brain activity underlying emotional processing from faces or voices in isolation (Campanella et al. 2010, 2012; Delle-Vigne et al. 2015).

We included two measures of trait anxiety as additional potential predictors in the models tested, to control for the effects of trait anxiety on intrusion development and to discern whether there was likely to be an interaction effect between multisensory predictors of intrusions and the anxiety measures. In contrast to previous findings using the trauma film paradigm, which have shown that an anxious emotional response to the trauma simulation predicted increased intrusions (Schweizer et al. 2017), neither of the anxiety measures used in this study emerged as significant predictors of intrusion frequency. Anxious arousal did emerge as a positive predictor of intrusion-related distress—i.e. higher anxious arousal predicted more distressing intrusive memories—but this effect failed to reach significance when using bootstrapped confidence intervals to estimate the model coefficients. Furthermore, no significant interaction effect between either of the anxiety measures and the multisensory performance measures was observed, which suggests that the observed multisensory effects on intrusion development were likely independent of the influence of trait anxiety.

Our findings could inform future studies investigating the role of multisensory processing in the development and treatment of PTSD. The finding that enhanced multisensory threat processing predicted greater intrusions suggests that cognitive bias modification (CBM) could be helpful in groups at-risk of PTSD to ameliorate emotional biases towards ‘over-processing’ of threat-related information and reduce the likelihood of developing intrusions (e.g. Woud et al. 2017). Our findings also suggest that CBM paradigms would benefit from incorporating multisensory stimuli, to specifically alter multisensory processing biases towards threat. In the current study, individuals who were more likely to be distracted by irrelevant emotional cues experienced fewer intrusions, which is in line with the argument made by Holmes et al. (2009) that performing visuospatial tasks around the time of trauma exposure could act as a ‘cognitive vaccine’ which protects against subsequent intrusion development by interfering with sensory-perceptual processing of the trauma. Overall, our study indicates that multisensory processing does play a role in the development of intrusions, which highlights the need for future research on multisensory processing in relation to PTSD. This is especially important as multisensory processing differences in PTSD could impact on the efficacy of treatment approaches that rely on multisensory stimulation, such as virtual reality exposure therapy (Asiain et al. 2021; Deng et al. 2019).

### Limitations

One of the limitations of the present study concerns the composition of the participant sample. The sample consisted largely of female university students, which raises issues of generalisability to the wider population, especially as sex differences have been demonstrated in multisensory processing of emotional information (Collignon et al. 2010). However, given that the prevalence of PTSD in women is approximately double the prevalence for men, investigating our research questions in a predominantly female sample is potentially more informative for understanding how traumatic events are processed by people who are at-risk of developing PTSD (Tolin and Foa 2008). Another limitation of the sample was that we were unable to recruit the target number of participants because of COVID-related laboratory closures in March 2020, which meant that the sample was only sufficiently powered to detect medium-to-large effects. Due to the limited sample size and the very stringent criteria used for entering predictors into the regression models, there may be smaller effects for less influential multisensory predictors that we have missed in our analyses, so there is more research to be done to uncover all of the potentially important effects. The study should be repeated with a larger sample size and a more focused set of predictors to increase the likelihood of capturing all the effects of multisensory processing on intrusion development.

For ethical reasons, we excluded those participants who were most likely to be excessively distressed by the VR simulation, such as individuals with a history of mental health problems, current low mood or PTSD symptoms. This is one of the reasons why we may have observed a smaller range of scores for intrusion frequency and intrusion-related distress compared to some previous studies (e.g. Belcher and Kangas 2015; Clark et al. 2015; Malik et al. 2014), which may have limited our ability to detect individual differences. Despite this, we still observed significant effects of our multisensory predictors. The exclusion of participants with prior vulnerability factors also limits how the results can be applied to intrusion development and maintenance in clinical PTSD populations, leaving this as an area for future research.

A further consideration for future studies would be to assess the potential role of perceived immersion during the trauma simulation in producing intrusions. Immersion may be defined as the extent to which an individual perceives themselves as being part of an environment which provides a continuous stream of experience (Witmer and Singer 1998). We did not assess immersion during the VR simulation in the current study, because this was not directly relevant to our main research questions about the role of multisensory processing, but a relationship between immersion during the trauma film simulation and intrusive symptoms has been demonstrated (Baptie et al. 2021). Therefore, future studies should account for the potential role of individual differences in immersion when assessing the impact of multisensory processes on intrusions.

Given preliminary evidence presented here that individual differences in multisensory emotional processing are related to intrusion development after trauma, future studies would benefit from specifically investigating whether multisensory processing of emotional information during the trauma simulation predicts subsequent intrusions. This was not done in the current study, as the aim here was to gather initial evidence to determine whether individual differences in multisensory processes may be related to intrusions after trauma, and so could potentially be used as a cognitive marker to indicate which people might be more likely to have an adverse psychological response after experiencing trauma. However, measuring multisensory emotion recognition within the trauma simulation would be a more direct way to ascertain whether peri-traumatic multisensory processing plays a role in shaping the psychological response to trauma.

### Conclusion

The findings showed that greater multisensory integration of threatening faces and voices predicted increased intrusions following exposure to a virtual reality trauma experience. However, there was also an effect of crossmodal interference which extended across all emotions, suggesting that greater susceptibility to distraction by irrelevant emotional cues, may be associated with a more adaptive psychological response to trauma. Our findings are the first to demonstrate a link between altered multisensory processing of emotion cues and frequency of intrusions after a traumatic event and suggest that differences in multisensory processing may be a more sensitive biomarker of psychological recovery after trauma than differences in processing information from single senses.

## Supplementary Information

Below is the link to the electronic supplementary material.

Online resource 1 (DOCX 597 kb)

## Data Availability

All data subjected to statistical analysis in this manuscript are openly available from the University of Exeter's institutional  repository at: 10.24378/exe.4584.
