# Supplementary material for: Multisensory processing of emotional cues predicts intrusive memories after virtual reality trauma
Source: Virtual Real. 2023 Apr 4;27(3):2043–57. doi: 10.1007/s10055-023-00784-1 (PMC10442266; doi:10.1007/s10055-023-00784-1)
Supplement: Supplementary file 1 — Online resource 1 (DOCX 597 kb) [file 10055_2023_784_MOESM1_ESM.docx]

**Multisensory Processing of Emotional Cues Predicts Intrusive Memories after Virtual Reality Trauma**

Naomi Heffer^a,b^, Emma Dennie^c^, Chris Ashwin^a,d^, Karin Petrini^a,e^ & Anke Karl^c^

^a^University of Bath, Department of Psychology, Bath, UK; ^b^Bath Spa University, School of Sciences, Bath, UK; ^c^University of Exeter, Mood Disorders Centre, Exeter, UK; ^d^ Centre for Applied Autism Research (CAAR), Bath, UK; ^e^The Centre for the Analysis of Motion, Entertainment Research and Applications (CAMERA), Bath, UK

**Corresponding Author:**

Naomi Heffer, PhD

Department of Psychology

University of Bath

Claverton Down

Bath. BA2 7AY

United Kingdom

Email: [n.r.heffer@bath.ac.uk](mailto:n.r.heffer@bath.ac.uk)

**Supplementary Material**

1. **Additional Methodological Details**

**Figure A1**

*Participant Flowchart*

**
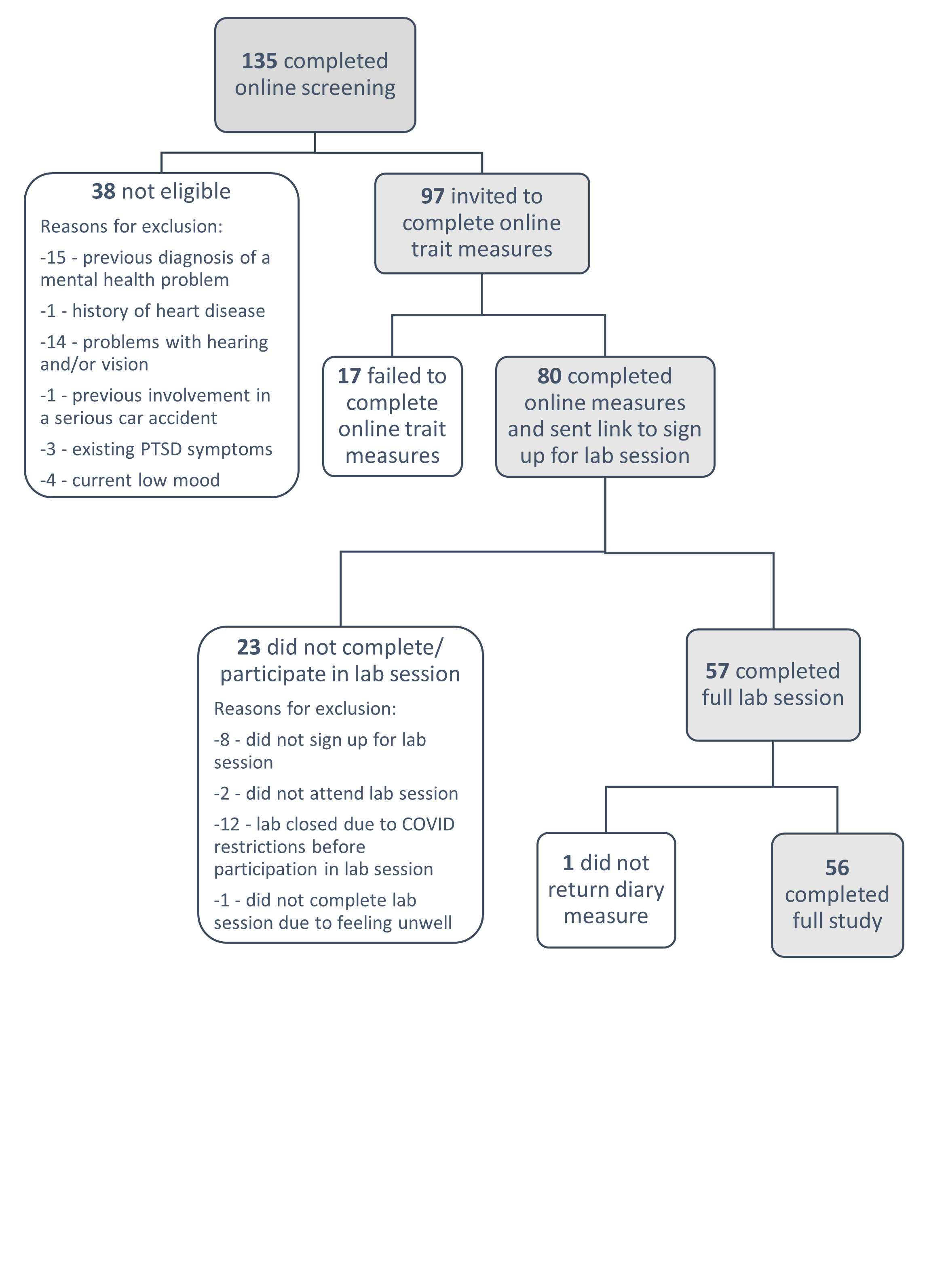
**

*Note*. The flowchart summarises participant recruitment and selection from initial screening to study completion.

**Trait Anxiety Measures**

***Spielberger Trait Anxiety Inventory***

The trait subscale of the Spielberger State-Trait Anxiety Inventory (STAI-T, Y-Form; Spielberger, 1983) was used to measure anxious apprehension. The trait subscale of the STAI consists of 20 items which assess the frequency of feelings relevant to “anxiety proneness” (1 almost never, 2 sometimes, 3 often, and 4 almost always), such as worry, confidence and security (e.g. “I feel secure”, “I feel nervous and restless”), with possible scores ranging from 20 to 80. The STAI-T has been shown to have strong psychometric properties, including high internal consistency, which was also the case in our sample (α = .92). The mean STAI-T score for participants in our sample was 37.77 (SD = 8.79), which is comparable to previously reported norm values for healthy adults (Bieling et al., 1998; Spielberger, 1983).

***Anxiety Sensitivity Index***

The ASI-3 is a short 18-item scale which measures fear of anxiety-related bodily sensations (Taylor et al. 2007) and has been recommended as a useful measure to isolate ‘anxious arousal’ processes from ‘anxious apprehension’ processes (Nitschke et al. 2001). The scale contains items which assess appraisals of physical anxiety symptoms (e.g. “It scares me when my heart beats rapidly”, “When I cannot keep my mind on a task, I worry that I might be going crazy”). Individuals rate how much they agree with each statement (0 very little, 1 little, 2 some, 3 much, and 4 very much) on the basis of their past experiences, or for items which concern something that they have never experienced (e.g. fainting in public), individuals answer on the basis of how they imagine they would feel if they ever had such an experience. In our sample, the overall scale had a Cronbach’s α of .90, indicative of high internal consistency. The mean ASI-3 score in our sample was 15.41 (SD = 10.94) which was comparable to previously reported values in the student population (Osman et al. 2010) and was below proposed cut-off’s for categorizing individuals as having ‘high’ or ‘moderate-to-high’ anxiety sensitivity (Allan et al. 2014).

1. **Behavioural Outcomes from Multisensory Emotion Recognition Tasks**

The multisensory emotion recognition tasks measured the ability to accurately recognize emotions from auditory, visual and audiovisual face and voice expressions. For an explanation of the three different tasks, the Unimodal, Undirected Attention and Directed Attention tasks, please see the methods section of the main manuscript. As well as the main accuracy measures, data from the task was also used to calculate measures of multisensory integration ability, crossmodal interference and emotional perceptual bias. Table 1 in the main manuscript shows all the different behavioural outcomes that were subjected to stepwise regression procedures as part of the analyses examining which factors related to multisensory emotional processing predicted psychological stress (i.e. intrusion frequency and distress related to intrusions) following exposure to the VR analogue trauma.

**Justification for Including Each of the Predictors**

Accuracy scores, i.e. the percentage of correct responses, were calculated separately for audio, visual and audiovisual congruent stimuli and for happy, angry, and sad stimuli, and were included as potential predictors to control for the potential impact of more general perceptual differences across individuals on intrusion development. Generalised difficulties with emotion recognition are associated with increased feelings of depression and anxiety (e.g. Kanne et al., 2009), and cognitive theories of PTSD suggest that the ability to adaptively process emotional cues at the time of the trauma is key to formation of structured trauma memories and psychological recovery after exposure to traumatic events (Brewin, 2001; Ehlers & Clark, 2000; Ehlers & Steil, 1995), therefore we could not rule out that low emotion recognition accuracy would be predictive of intrusions after exposure to VR trauma. We included accuracy scores for audio, visual and audiovisual stimuli as separate predictors in order to separate out the potential role of general difficulties in emotion recognition extending across sensory modalities, from specific issues recognizing emotion from multisensory stimuli, which requires additional integration processes relative to processing of unimodal emotional information (Klasen et al. 2012; Schirmer & Adolphs, 2017) which have been shown to be disrupted in some clinical populations (De Jong et al. 2009; Feldman et al. 2018; Koizumi et al. 2011; Maurage & Campanella, 2013). We also included accuracy scores separately for each emotion, as processing advantages for negative emotional cues, such as sad faces and voices, and threat-related emotional cues, such as angry faces and voices, have also been associated with negative psychological outcomes (e.g. Bourke et al., 2010; Cisler & Koster, 2010). We included a separate measure of accuracy for happy stimuli as affective problems, such as symptoms associated with depression and anxiety, have not only been associated with a bias towards over-processing negative/threat signals, but also a reduced tendency to orient towards and focus on positive emotional cues (e.g. Chen et al. 2012, 2016; Taylor et al. 2010), so it was hypothesized that reduced accuracy for perception of happy stimuli could be predictive of increased intrusions in the current study.

In line with the justification above for including separate measures of accuracy for each emotion, we also calculated multiple predictors involving a ‘Multisensory Facilitation Score’, both as an overall measure of multisensory integration ability, which has been shown to be disrupted in some clinical populations as outlined above, and separately for each emotion, to determine whether multisensory processing advantages or disadvantages for positive, negative or threat-related emotions specifically, might influence the frequency and intensity of intrusive memories.

For audiovisual incongruent trials in the Undirected Attention Task, we calculated Multisensory Threat Bias and Multisensory Negativity Bias outcomes, which we hypothesised might be important as selective multisensory integration of negative or threat-relevant information, as has been observed in high trait anxiety (Heffer et al. 2021, 2022), could lead to enhanced threat-processing of stressful life events and result in increased intrusions (Mancini et al. 2021). We calculated separate outcomes for threat-related bias compared to negativity bias to allow us to separate out general negativity effects, typically seen in depression (Bourke et al. 2010) from threat-specific biases, typically seen in anxiety (Cisler & Koster, 2010).

Finally, we also calculated ‘Crossmodal Interference’ from the Directed Attention tasks, which gives a measure of the level of perceptual interference caused by to-be-ignored face or voice cues in audiovisual incongruent displays. In the same way as for the Multisensory Facilitation Scores, Crossmodal Interference was calculated overall across all stimulus emotions, and then separately for each of the three emotions. The rationale for doing so is similar to what has already been presented, but in short, overall crossmodal interference has been shown to be associated with poorer executive control (Araneda et al. 2015; Hirst et al. 2019; Spagna et al. 2020), which has previously been identified as a predictor of increased intrusions following the trauma film paradigm (Verwoerd et al. 2011). Crossmodal interference resulting specifically from processing of irrelevant angry information has been linked to trait anxiety (Koizumi et al. 2011), and so we also had reason to believe that differences in this predictor, specifically for angry stimuli, might also predict increased intrusions.

**Analysis of Multisensory Task Performance**

Descriptive statistics relating to group performance on the different behavioural measures are summarised in Tables B1 – B7. The numbers assigned to the variables are the same as the numbers given in Table 1 of the main manuscript where the measures are first described. Non-parametric descriptive and inferential statistics are reported for the outcomes where the distribution of scores was found to deviate significantly from the normal distribution.

**Table B1**

*Accuracy in the Unimodal and Undirected Attention Tasks as a Function of Stimulus Modality*

|  | Stimulus Modality | Median (IQR) Accuracy (%) |
| --- | --- | --- |
| 1. | Audio-only | 81.48 (16.67) |
| 2. | Visual-only | 68.52 (11.11) |
| 3. | AVC | 81.48 (12.96) |

*Note:* AVC = audiovisual congruent

Consistent with the descriptive statistics summarised in Table B1, the results of two Wilcoxon tests showed that participants were significantly more accurate on Audiovisual Congruent trials of the Undirected Attention task compared to Visual-only trials of the unimodal task, *Z* = -6.00, *p* < .001, *d* = 1.01, but were not significantly more accurate on Audiovisual Congruent trials compared to Audio-only trials, *Z* = -0.35, *p* = .725.

**Table B2**

*Accuracy in the Unimodal and Undirected Attention Tasks as a Function of Stimulus Emotion*

|  | Target Emotion | Median (IQR) Accuracy (%) |
| --- | --- | --- |
| 4. | Happy | 79.63 (14.81) |
| 5. | Angry | 79.63 (12.96) |
| 6. | Sad | 70.37 (22.22) |

**Table B3**

*Multisensory Facilitation Scores based on Accuracy in the Unimodal and Undirected Attention Tasks*

|  | Target Emotion | Mean (SD) Facilitation Score |
| --- | --- | --- |
| 7. | Overall | -1.01 (8.10) |
| 8. | Happy | -7.58 (11.48) |
| 9. | Angry | -0.30 (12.40) |
| 10. | Sad | -4.44 (13.87) |

*Note:* Facilitation Score = difference between accuracy in the audiovisual congruent (AVC) condition and that in the best unimodal condition, i.e., Facilitation Score = p(AVC) - max{p(A), p(V)}, where p is the percentage of correct responses in each condition. Positive scores indicate multisensory gain and negative scores indicate better performance with only one of the senses.

A one-tailed t-test showed that the Overall Facilitation Score was not significantly different from zero, *t* (54) = -0.93, *p* = .359, 95% CI [-3.20, 1.18]. This indicates that there was no significant multisensory benefit to emotion recognition accuracy in the audiovisual congruent condition of the Undirected Attention Task compared to performance in the Unimodal Task.

**Table B4**

*Multisensory Bias for Audiovisual Incongruent Trials of the Undirected Attention Task*

|  | Type of Bias | Mean (SD) |
| --- | --- | --- |
| 11. | Threat Bias | 2.46 (8.94) |
| 12. | Negativity Bias | -0.01 (11.26) |

*Note:* A score of 0 indicates no bias, positive scores indicate a bias towards threatening/negative interpretations of stimuli, negative scores indicate a bias away from threatening/negative interpretations. Threat bias was calculated as the percentage of ‘angry’ judgements on audiovisual incongruent trials minus the expected percentage for a neutral observer (i.e. 33.33%). Negativity bias was calculated as the percentage of ‘sad’ and ‘angry’ judgements on audiovisual incongruent trials minus the expected percentage for a neutral observer (i.e. 66.67%).

Consistent with the descriptive statistics summarised in Table B4, the results of two one-sample t-tests comparing the mean bias scores to zero showed that participants exhibited a small but significant bias towards threat (i.e. they were more likely to perceive stimuli as angry) when judging emotion from audiovisual incongruent stimuli in the Undirected Attention task, *t* (54) = 2.04, *p* = .046, 95% CI [0.04, 4.88], *d* = 0.28. However, participants did not exhibit a significant bias towards or away from negative interpretations of stimuli (i.e. sad and angry) more generally, *t* (54) = -0.01, *p* = .996, 95% CI [-3.05, 3.04].

An alternative way of calculating the multisensory bias outcomes was proposed, whereby bias scores are calculated based only on the stimulus material that is presented in each audiovisual incongruent stimulus combination. So, negativity bias is calculated as the tendency to respond ‘sad’ to sad/happy combination stimuli above a chance level of 50%, and threat bias is calculated as the tendency to respond ‘angry’ to angry/happy combination stimuli above a chance level of 50%. Table B5 shows the mean values for the multisensory bias outcomes when calculated in this manner.

**Table B5**

*Alternative Multisensory Bias Scores for Audiovisual Incongruent Trials of the Undirected Attention Task*

| Type of Bias | Mean (SD) |
| --- | --- |
| Threat Bias | -6.06 (11.58) |
| Negativity Bias | -12.83 (13.70) |

*Note:* A score of 0 indicates no bias, positive scores indicate a bias towards threatening/negative interpretations of stimuli, negative scores indicate a bias away from threatening/negative interpretations.

Consistent with the data summarised in Table B5, the results of two one-sample t-tests comparing the mean bias scores to zero showed that participants exhibited a significant bias towards positive interpretations of audiovisual incongruent stimuli (i.e. they were more likely to perceive stimuli as happy) when judging emotion from audiovisual incongruent stimuli involving happy and angry cues, *t* (54) = -3.88, *p* < .001, 95% CI [-9.19, -2.93], *d* = -0.52, and when judging emotion from audiovisual incongruent stimuli involving happy and sad cues *t* (54) = -6.95, *p* < .001, 95% CI [-16.53, -9.13], *d* = -0.94.

**Table B6**

*Percentage judgements for each emotion for Audiovisual Incongruent Trials of the Undirected Attention Task*

|  |  | % Judgements for each emotion | | |
| --- | --- | --- | --- | --- |
| Face emotion | Voice emotion | Angry | Happy | Sad |
| Angry | Happy | 35.56 | 43.43 | **21.01** |
|  | Sad | 25.25 | **14.04** | 60.71 |
| Happy | Angry | 52.32 | 39.90 | **7.68** |
|  | Sad | **11.01** | 47.37 | 41.62 |
| Sad | Angry | 73.23 | **5.35** | 21.41 |
|  | Happy | **17.37** | 49.90 | 32.73 |

The results presented in Table B6 show that even when an emotion is not represented in either the audio or visual component of the face and voice stimulus, participants may still perceive it. This shows evidence of an emotional McGurk effect (Fagel, 2006), where integration of incongruent cues (such as an angry face with a happy voice) results in perception of an entirely different emotion (i.e. sad). The results in the table which show evidence of an emotional McGurk effect are formatted in bold.

**Table B7**

*Crossmodal Interference in the Directed Attention Task as a Function of the Emotion Portrayed in the Unattended Cue*

|  | Distractor Emotion | Median (IQR) Crossmodal Interference Effect |
| --- | --- | --- |
| 13. | Overall | 18.98 (17.59) |
| 14. | Happy | 19.91 (20.37) |
| 15. | Angry | 20.83 (22.69) |
| 16. | Sad | 19.91 (23.61) |

*Note:* crossmodal interference was calculated by subtracting percentage accuracy in the audiovisual incongruent trials from percentage accuracy in the audiovisual congruent trials for each participant. Larger interference effects indicate that processing of incongruent information in the to-be-ignored modality was more likely to interfere with the perception of emotion for information in the attended modality.

Consistent with the descriptive statistics summarised in Table B7, a Wilcoxon signed-rank test showed that the Overall Crossmodal Interference Effect was significantly greater than zero, *Z* = 6.45, *p* < .001, *d* =1.08. This indicates that in the Directed Attention Task participants were significantly less accurate at recognising emotions from cues in the target modality when these were presented alongside emotionally incongruent ‘distractor’ cues, compared to when target cues were presented alongside additional congruent emotional cues.

**Table B8**

*Correlation Matrix for all Predictor Variables*

|  | A Acc | V Acc | AV  Acc | Happy  Acc | Angry  Acc | Sad Acc | MF | Happy MF | Angry MF | Sad MF | CM | Happy CM | Angry CM | Sad CM | Threat Bias | Negative Bias | STAI | ASI-3 |
| --- | --- | --- | --- | --- | --- | --- | --- | --- | --- | --- | --- | --- | --- | --- | --- | --- | --- | --- |
| A Acc | 1.00 | 0.38 | 0.41 | 0.52 | 0.20 | 0.50 | -0.66 | -0.23 | -0.13 | -0.29 | 0.11 | 0.17 | -0.05 | 0.20 | -0.25 | -0.01 | -0.21 | -0.04 |
| V Acc | 0.38 | 1.00 | 0.43 | 0.23 | 0.57 | 0.49 | -0.15 | -0.18 | -0.02 | -0.01 | 0.27 | 0.07 | 0.23 | 0.26 | 0.17 | 0.35 | -0.09 | -0.03 |
| AV Acc | 0.41 | 0.43 | 1.00 | 0.17 | 0.30 | 0.66 | 0.33 | 0.14 | 0.31 | 0.48 | 0.34 | 0.13 | 0.16 | 0.45 | 0.06 | 0.36 | -0.16 | 0.08 |
| Happy Acc | 0.52 | 0.23 | 0.17 | 1.00 | -0.23 | 0.01 | -0.41 | 0.02 | 0.03 | -0.40 | 0.15 | 0.50 | -0.11 | 0.03 | -0.32 | -0.40 | -0.05 | -0.13 |
| Angry Acc | 0.20 | 0.57 | 0.30 | -0.23 | 1.00 | 0.05 | 0.02 | -0.06 | -0.06 | 0.07 | 0.08 | -0.18 | 0.35 | -0.01 | 0.60 | 0.43 | 0.02 | 0.14 |
| Sad Acc | 0.50 | 0.49 | 0.66 | 0.01 | 0.05 | 1.00 | -0.04 | -0.18 | 0.15 | 0.30 | 0.24 | -0.05 | -0.02 | 0.54 | -0.19 | 0.46 | -0.17 | 0.02 |
| MF | -0.66 | -0.15 | 0.33 | -0.41 | 0.02 | -0.04 | 1.00 | 0.41 | 0.43 | 0.66 | 0.05 | -0.15 | 0.10 | 0.07 | 0.36 | 0.25 | 0.03 | 0.04 |
| Happy MF | -0.23 | -0.18 | 0.14 | 0.02 | -0.06 | -0.18 | 0.41 | 1.00 | -0.09 | 0.07 | 0.03 | 0.12 | -0.03 | -0.07 | 0.05 | -0.26 | -0.09 | -0.08 |
| Angry MF | -0.13 | -0.02 | 0.31 | 0.03 | -0.06 | 0.15 | 0.43 | -0.09 | 1.00 | 0.08 | -0.02 | -0.06 | -0.01 | 0.07 | 0.24 | 0.14 | 0.05 | 0.04 |
| Sad MF | -0.29 | -0.01 | 0.48 | -0.40 | 0.07 | 0.30 | 0.66 | 0.07 | 0.08 | 1.00 | 0.08 | -0.20 | 0.02 | 0.23 | 0.13 | 0.41 | -0.03 | 0.01 |
| CM | 0.11 | 0.27 | 0.34 | 0.15 | 0.08 | 0.24 | 0.05 | 0.03 | -0.02 | 0.08 | 1.00 | 0.74 | 0.76 | 0.83 | -0.09 | 0.20 | 0.13 | -0.02 |
| Happy CM | 0.17 | 0.07 | 0.13 | 0.50 | -0.18 | -0.05 | -0.15 | 0.12 | -0.06 | -0.20 | 0.74 | 1.00 | 0.42 | 0.47 | -0.33 | -0.31 | 0.11 | -0.07 |
| Angry CM | -0.05 | 0.23 | 0.16 | -0.11 | 0.35 | -0.02 | 0.10 | -0.03 | -0.01 | 0.02 | 0.76 | 0.42 | 1.00 | 0.48 | 0.31 | 0.35 | 0.42 | 0.17 |
| Sad CM | 0.20 | 0.26 | 0.45 | 0.03 | -0.01 | 0.54 | 0.07 | -0.07 | 0.07 | 0.23 | 0.83 | 0.47 | 0.48 | 1.00 | -0.22 | 0.31 | -0.04 | -0.04 |
| Threat Bias | -0.25 | 0.17 | 0.06 | -0.32 | 0.60 | -0.19 | 0.36 | 0.05 | 0.24 | 0.13 | -0.09 | -0.33 | 0.31 | -0.22 | 1.00 | 0.51 | 0.17 | 0.16 |
| Negative Bias | -0.01 | 0.35 | 0.36 | -0.40 | 0.43 | 0.46 | 0.25 | -0.26 | 0.14 | 0.41 | 0.20 | -0.31 | 0.35 | 0.31 | 0.51 | 1.00 | 0.03 | 0.15 |
| STAI | -0.21 | -0.09 | -0.16 | -0.05 | 0.02 | -0.17 | 0.03 | -0.09 | 0.05 | -0.03 | 0.13 | 0.11 | 0.42 | -0.04 | 0.17 | 0.03 | 1.00 | 0.56 |
| ASI-3 | -0.04 | -0.03 | 0.08 | -0.13 | 0.14 | 0.02 | 0.04 | -0.08 | 0.04 | 0.01 | -0.02 | -0.07 | 0.17 | -0.04 | 0.16 | 0.15 | 0.56 | 1.00 |

*Note.* Correlation coefficients are Spearman’s Rho. A non-parametric correlation test was used as we did not necessarily expect a linear relationship between variables. Although correlation coefficient values between some variables are quite high, we used a stepwise procedure for entering regressors into the main analyses, so only a small number of variables which explained the greatest variance in the outcome variables were included in the final models, therefore, collinearity was not a problem. A = Audio; V = Visual; AV = Audiovisual; Acc = Accuracy; MF = Multisensory facilitation; CM = Crossmodal interference.

1. **Intrusion Frequency: Goodness of Fit and Generalisability Statistics**

**Figure C1**

*Histogram showing Distribution of Intrusion Frequency Outcome*

**
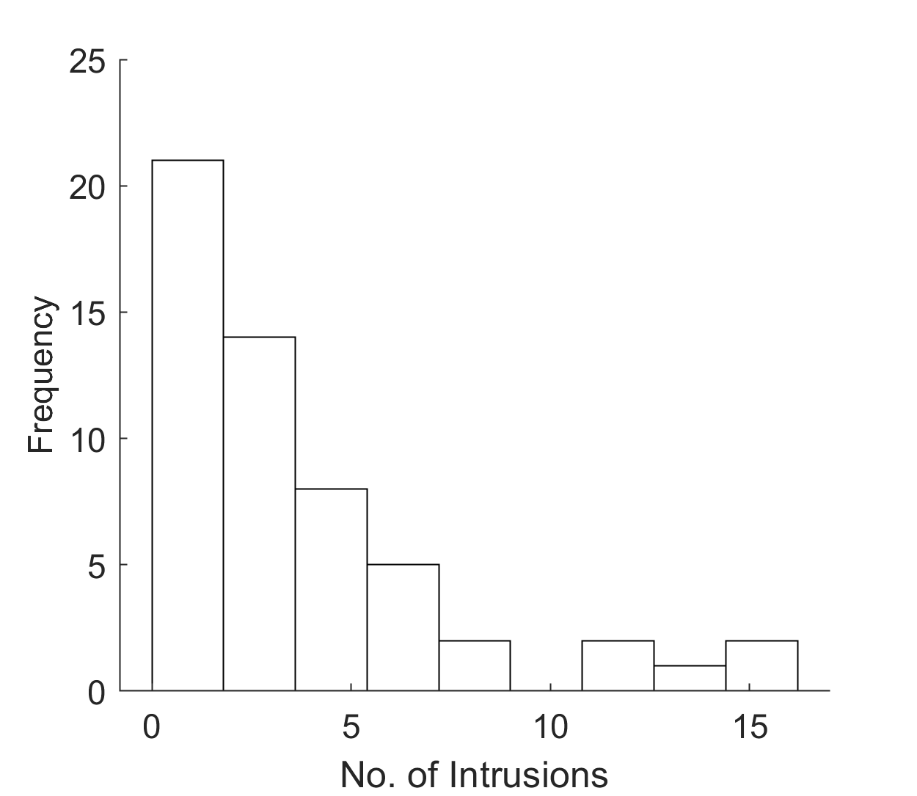
**

**Assessing Influential Cases**

In order to assess the fit of our final negative binomial model, which we obtained by stepwise procedures, we identified cases which had large studentized residuals (≥|2|). We identified one case a with studentized residuals that exceeded ±2. This case had a Cooks Distance of less than 0.5, which suggests that despite having a large residual, it was not exerting undue influence on the regression (Cook & Weisberg, 1982).

**Checking Residuals**

Next, we plotted the obtained residuals against the predicted values in order to check for linearity and homoscedasticity of the residuals. As can be seen from Figure C2, the relationship between obtained residuals and predicted values was approximately linear and residuals appeared largely homoscedastic.

We assessed the extent to which the obtained residuals approximated the normal distribution by plotting studentized residuals as a histogram and running a Shapiro-Wilk test. The shape of the histogram shown in Figure C2 and the results of the Shapiro-Wilk test, *W* = 0.98, *p* = .345, suggested that the obtained residuals were approximately normally distributed.

**Figure C2**

*Residual Plots for the Negative Binomial Regression Model*


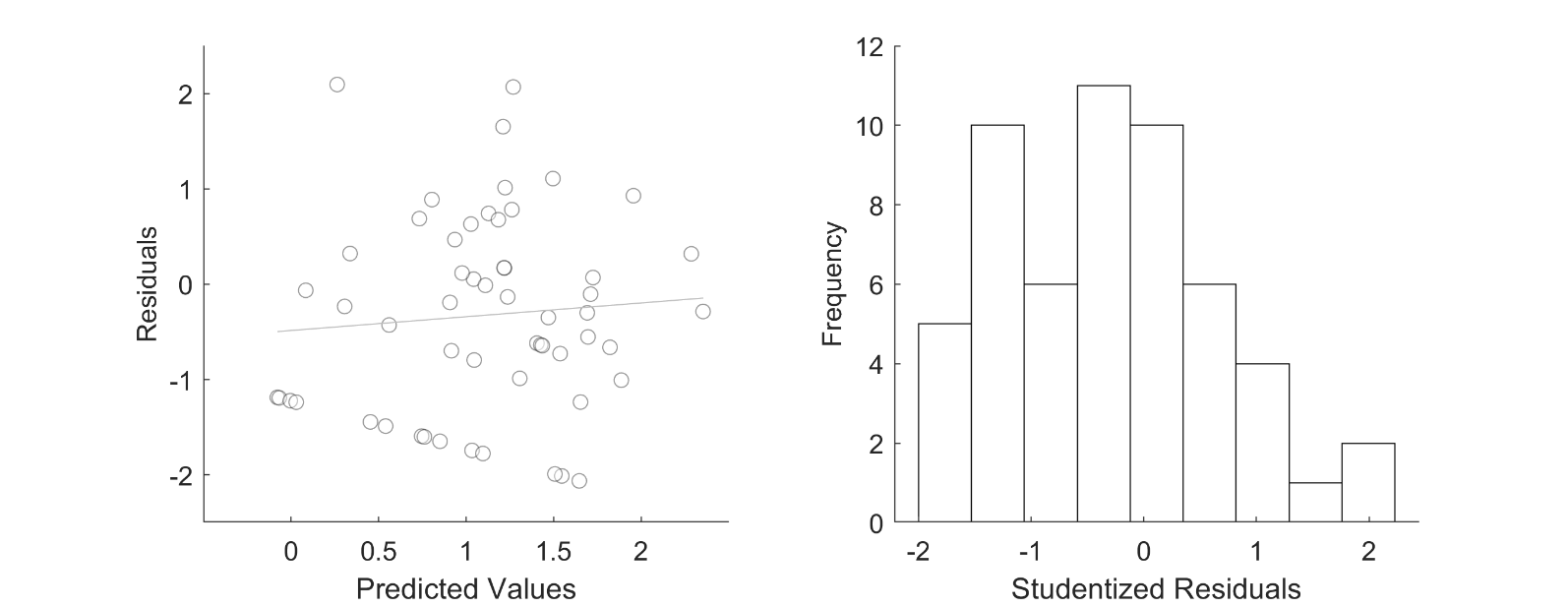


*Note.* Residual plots demonstrating goodness of fit of the negative binomial regression used to model the intrusion frequency data: Number of Intrusions ~ Angry Facilitation Score + Crossmodal Interference + Sad Accuracy. Panel (a) shows predicted values plotted against the obtained residuals, with a linear regression line added. Panel (b) is a histogram showing the distribution of studentized residuals.

**Checking for Multicollinearity**

The VIF and tolerance values presented in Table C1 indicate that multicollinearity did not exceed acceptable levels. Generally, there is considered to be a problem with multicollinearity only if the largest VIF value exceeds ten, or if the average VIF value is substantially greater than one (Bowerman & O'Connell, 1990; Myers, 1990), neither of which was the case here. Tolerance values below 0.2 can also indicate potential bias in the model due to multicollinearity (Menard, 1995), but again, this was not a problem here.

**Table C1**

*VIF Values and Tolerance Values used to Assess Potential Multicollinearity in the Model*

| Predictor | VIF Value | Tolerance Value |
| --- | --- | --- |
| Angry Facilitation Score | 1.02 | 0.98 |
| Crossmodal Interference | 1.07 | 0.94 |
| Sad Accuracy | 1.08 | 0.92 |

**D. Distress Related to Intrusions: Goodness of Fit and Generalisability Statistics**

**Figure D1**

*Histogram showing Distribution of Ratings of Intrusion-Related Distress*

**
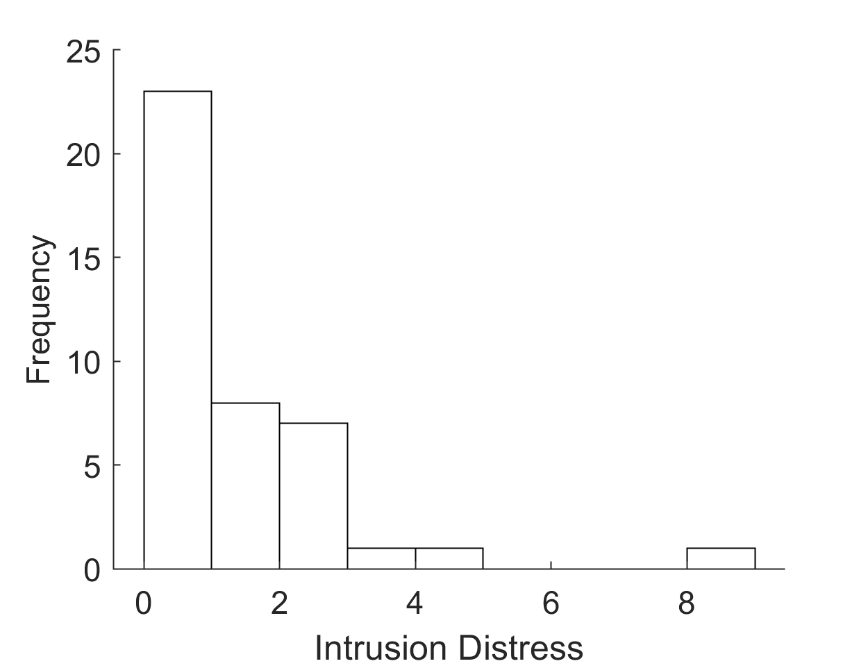
**

*Note.* The figure shows the mean level of daily distress reported by participants who experienced at least one intrusion (*n* = 41). For details of how this was calculated, see the methods section of the main manuscript.

**Assessing Influential Cases**

In order to assess the fit of our beta regression model, which we obtained by stepwise procedures, we identified cases which had large studentized residuals (≥|2|). We identified one case with a studentized residual that exceeded ±2. In addition to having a large residual, this case also had a large Cook’s distance (0.80), which suggested that it was likely to be exerting undue influence on the regression. As a result of this we reran the model while winsorizing this case (replacing the outcome value with the next most extreme value in the dataset), and these are the results which are presented in the main results section (Table 3). The results from the model before this outlier was winsorized are presented in Table D1.

When the model was rerun with the influential outlier winsorized, we identified two cases with a studentized residual that exceeded ±2, but both had a Cooks Distance of less than 0.5, which suggests that despite having a large residual, these cases were not exerting undue influence on the regression (Cook & Weisberg, 1982).

**Table D1**

*Distress Related to Intrusions Stepwise Regression Model before Winsorizing Influential Outlier*

| **Step** | **Variable** | **Pseudo R^2^** | **B** | **SE** | **p** |
| --- | --- | --- | --- | --- | --- |
| 1 | Constant | 0.18 | -1.03 | 0.28 | <.001 |
|  | Crossmodal Interference |  | -3.45 | 1.29 | .008 |
| 2 | Constant | 0.29 | -1.45 | 0.34 | <.001 |
|  | Crossmodal Interference |  | -3.13 | 1.26 | .013 |
|  | Anxious Arousal (ASI-3 Score) |  | 0.02 | 0.01 | .036 |

**Checking Residuals**

Next, we plotted the obtained residuals against the predicted values in order to check for linearity and homoscedasticity of the residuals. As can be seen from Figure D2, the relationship between obtained residuals and predicted values was approximately linear and residuals appeared largely homoscedastic.

We assessed the extent to which the obtained residuals approximated the normal distribution by plotting studentized residuals as a histogram and running a Shapiro-Wilk test. The shape of the histogram shown in Figure D1 and the results of the Shapiro-Wilk test, *W* = 0.90, *p* = .002, suggested that the obtained residuals were non-normally distributed. As we cannot assume that the underlying distribution of the data is normal, bootstrapped estimates for the value of predictor coefficients should be preferentially interpreted.

**Figure D2**

*Residual Plots for the Beta Regression Model*
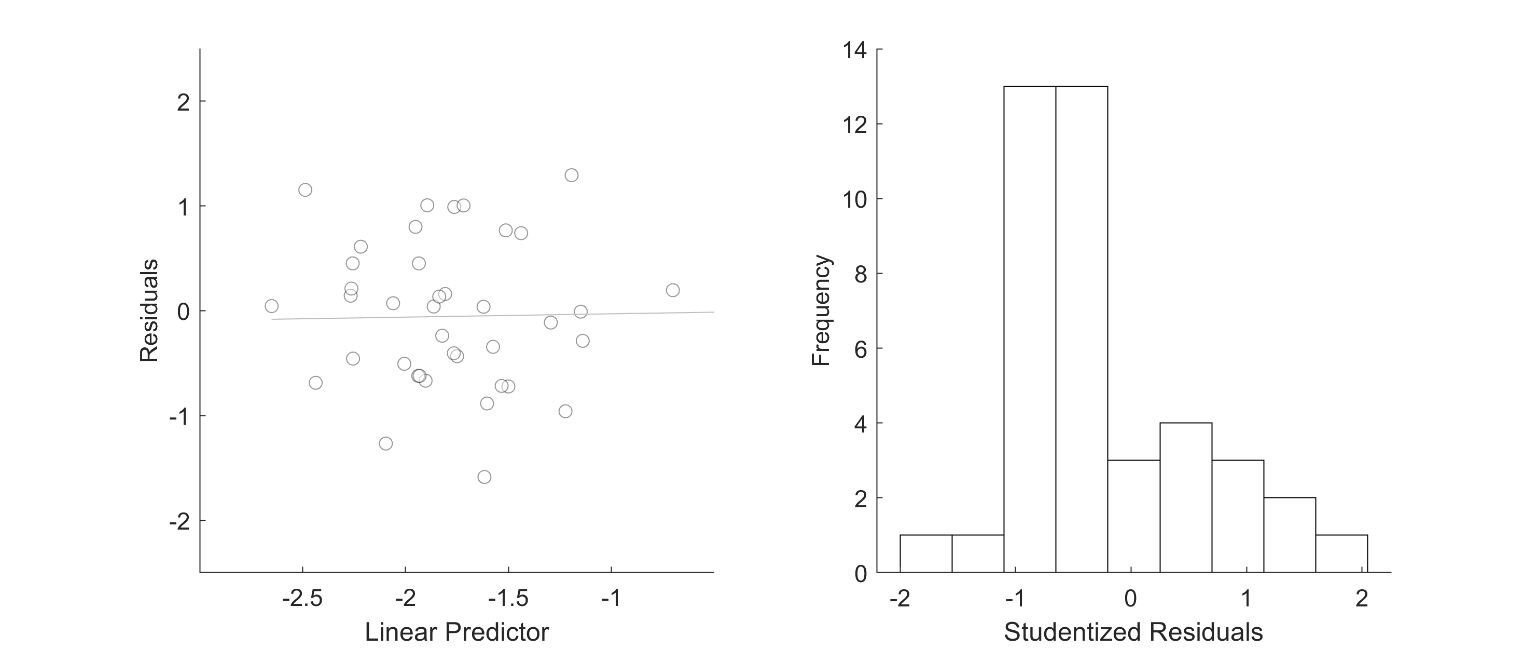


*Note.* Residual plots demonstrating goodness of fit of the beta regression used to model the intrusion distress data: Mean Distress Rating ~ Crossmodal Interference + Anxious Arousal. Panel (a) shows linear predicted values plotted against the obtained residuals, with a linear regression line added. Panel (b) is a histogram showing the distribution of studentized residuals.

**Checking for Multicollinearity**

The VIF and tolerance values presented in Table D2 indicate that multicollinearity did not exceed acceptable levels. Generally, there is considered to be a problem with multicollinearity only if the largest VIF value exceeds ten, or if the average VIF value is substantially greater than one (Bowerman & O'Connell, 1990; Myers, 1990), neither of which was the case here. Tolerance values below 0.2 can also indicate potential bias in the model due to multicollinearity (Menard, 1995), but again, this was not a problem here.

**Table D2**

*VIF Values and Tolerance Values used to Assess Potential Multicollinearity in the Model*

| Predictor | VIF Value | Tolerance Value |
| --- | --- | --- |
| Crossmodal Interference | 1.06 | 0.95 |
| Anxious Arousal | 1.05 | 0.95 |

**References**

Allan, N. P., Raines, A. M., Capron, D. W., Norr, A. M., Zvolensky, M. J., & Schmidt, N. B. (2014). Identification of anxiety sensitivity classes and clinical cut-scores in a sample of adult smokers: Results from a factor mixture model. *Journal of Anxiety Disorders*, *28*(7), 696–703. https://doi.org/10.1016/j.janxdis.2014.07.006

Araneda, R., Volder, A. G. D., Deggouj, N., & Renier, L. (2015). Altered inhibitory control and increased sensitivity to cross-modal interference in tinnitus during auditory and visual tasks. *PLOS ONE*, *10*(3), e0120387. https://doi.org/10.1371/journal.pone.0120387

Bieling, P. J., Antony, M. M., & Swinson, R. P. (1998). The State–Trait Anxiety Inventory, Trait version: Structure and content re-examined. *Behavior Research and Therapy*, *36*(7–8), 777–788. https://doi.org/10.1016/S0005-7967(98)00023-0

Bourke, C., Douglas, K., & Porter, R. (2010). Processing of facial emotion expression in major depression: A review. *Australian & New Zealand Journal of Psychiatry*, *44*(8), 681–696. https://doi.org/10.3109/00048674.2010.496359

Bowerman, B. L., & O'Connell, R. T. (1990). *Linear statistical models: An applied approach.* Brooks/Cole.

Brewin, C. R. (2001). Memory processes in post-traumatic stress disorder. *International Review of Psychiatry*, *13*(3), 159–163. https://doi.org/10.1080/09540260120074019

Chen, N. T. M., Clarke, P. J. F., MacLeod, C., & Guastella, A. J. (2012). Biased attentional processing of positive stimuli in social anxiety disorder: An eye movement study. *Cognitive Behaviour Therapy, 41*(2), 96–107. https://doi.org/10.1080/16506073.2012.666562

Chen, N. T. M., Clarke, P. J. F., MacLeod, C., Hickie, I. B., & Guastella, A. J. (2016). Aberrant gaze patterns in social anxiety disorder: An eye movement assessment during public speaking. *Journal of Experimental Psychopathology, 7*(1), 1–17. https://doi.org/10.5127/jep.040313

Cisler, J. M., & Koster, E. H. W. (2010). Mechanisms of attentional biases towards threat in the anxiety disorders: An integrative review. *Clinical Psychology Review, 30*(2), 203. https://doi.org/10.1016/j.cpr.2009.11.003

Cook, R. D., & Weisberg, S. (1982). *Residuals and influence in regression*. Chapman and Hall

De Jong, J. J., Hodiamont, P. P. G., Van den Stock, J., & De Gelder, B. (2009). Audiovisual emotion recognition in schizophrenia: Reduced integration of facial and vocal affect. *Schizophrenia Research*, *107*(2–3), 286–293. https://doi.org/10.1016/j.schres.2008.10.001

Ehlers, A., & Clark, D. M. (2000). A cognitive model of posttraumatic stress disorder. *Behaviour Research and Therapy*, *38*(4), 319–345. https://doi.org/10.1016/S0005-7967(99)00123-0

Ehlers, A., & Steil, R. (1995). Maintenance of intrusive memories in posttraumatic stress disorder: A cognitive approach. *Behavioural and Cognitive Psychotherapy*, *23*(3), 217–249. https://doi.org/10.1017/S135246580001585X

Fagel, S. (2006). Emotional McGurk effect. *Proceedings of the International Conference on Speech Prosody*, *1*.

Feldman, J. I., Dunham, K., Cassidy, M., Wallace, M. T., Liu, Y., & Woynaroski, T. G. (2018). Audiovisual multisensory integration in individuals with autism spectrum disorder: A systematic review and meta-analysis. *Neuroscience & Biobehavioral Reviews*, *95*, 220–234. https://doi.org/10.1016/j.neubiorev.2018.09.020

Hirst, R. J., Kicks, E. C., Allen, H. A., & Cragg, L. (2019). Cross-modal interference-control is reduced in childhood but maintained in aging: A cohort study of stimulus-and response-interference in cross-modal and unimodal Stroop tasks. *Journal of Experimental Psychology: Human Perception and Performance*, *45*(5), 553. https://doi.org/10.1037/xhp0000608

Kanne, S. M., Christ, S. E., & Reiersen, A. M. (2009). Psychiatric symptoms and psychosocial difficulties in young adults with autistic traits. *Journal of Autism and Developmental Disorders, 39*(6), 827–833. https://doi.org/10.1007/s10803-008-0688-x

Klasen, M., Chen, Y.-H., & Mathiak, K. (2012). Multisensory emotions: Perception, combination and underlying neural processes. *Reviews in the Neurosciences*, *23*(4), 381–392. https://doi.org/10.1515/revneuro-2012-0040

Koizumi, A., Tanaka, A., Imai, H., Hiramatsu, S., Hiramoto, E., Sato, T., & de Gelder, B. (2011). The effects of anxiety on the interpretation of emotion in the face–voice pairs. *Experimental Brain Research*, *213*(2), 275–282. https://doi.org/10.1007/s00221-011-2668-1

Mancini, A. D., Aldrich, L., Shevorykin, A., Veith, S., & John, G. (2021). Threat appraisals, neuroticism, and intrusive memories: A robust mediational approach with replication. *Anxiety, Stress, & Coping*, *34*(1), 66–82. https://doi.org/10.1080/10615806.2020.1825693

Maurage, P., & Campanella, S. (2013). Experimental and clinical usefulness of crossmodal paradigms in psychiatry: An illustration from emotional processing in alcohol-dependence. *Frontiers in Human Neuroscience*, *7,* 394. https://doi.org/10.3389/fnhum.2013.00394

Menard, S. W. (1995). *Applied logistic regression analysis*. Sage.

Meredith, M. A., & Stein, B. E. (1983). Interactions among converging sensory inputs in the superior colliculus. *Science*, *221*(4608), 389–391. https://doi.org/10.1126/science.6867718

Myers, R. H. (1990). *Classical and modern regression with applications*. Duxbury press.

Nitschke, J. B., Heller, W., Imig, J. C., McDonald, R. P., & Miller, G. A. (2001). Distinguishing dimensions of anxiety and depression. *Cognitive Therapy and Research*, *25*(1), 1–22. https://doi.org/10.1023/A:1026485530405

Osman, A., Gutierrez, P. M., Smith, K., Fang, Q., Lozano, G., & Devine, A. (2010). The Anxiety Sensitivity Index–3: Analyses of dimensions, reliability estimates, and correlates in nonclinical samples. *Journal of Personality Assessment*, *92*(1), 45–52. https://doi.org/10.1080/00223890903379332

Schirmer, A., & Adolphs, R. (2017). Emotion perception from face, voice, and touch: Comparisons and convergence. *Trends in Cognitive Sciences*, *21*(3), 216–228. https://doi.org/10.1016/j.tics.2017.01.001

Spagna, A., Wu, T., Kim, K., & Fan, J. (2020). Supramodal executive control of attention: Evidence from unimodal and crossmodal dual conflict effects. *Cortex*, *133*, 266–276. https://doi.org/10.1016/j.cortex.2020.09.018

Spielberger, C. D., Gorsuch, R.L., Lushene, R., Vagg, P.R., Jacobs, G.A. (1983). *Manual for the State–Trait Anxiety Inventory, STAI (Form Y): Self-evaluation questionnaire.* Consulting Psychologists Press.

Taylor, C. T., Bomyea, J., & Amir, N. (2010). Attentional bias away from positive social information mediates the link between social anxiety and anxiety vulnerability to a social stressor. *Journal of Anxiety Disorders, 24*(4), 403–408. https://doi.org/10.1016/j.janxdis.2010.02.004

Taylor, S., Zvolensky, M. J., Cox, B. J., Deacon, B., Heimberg, R. G., Ledley, D. R., Abramowitz, J. S., Holaway, R. M., Sandin, B., & Stewart, S. H. (2007). Robust dimensions of anxiety sensitivity: Development and initial validation of the Anxiety Sensitivity Index-3. *Psychological Assessment*, *19*(2), 176. https://doi.org/10.1037/1040-3590.19.2.176

Verwoerd, J., Wessel, I., De Jong, P. J., Nieuwenhuis, M. M., & Huntjens, R. J. (2011). Pre-stressor interference control and intrusive memories. *Cognitive Therapy and Research*, *35*(2), 161–170. https://doi.org/10.1007/s10608-010-9335-x
